# Diversity of echinostomes (Digenea: Echinostomatidae) in their snail hosts at high latitudes

**DOI:** 10.1051/parasite/2021054

**Published:** 2021-07-28

**Authors:** Camila Pantoja, Anna Faltýnková, Katie O’Dwyer, Damien Jouet, Karl Skírnisson, Olena Kudlai

**Affiliations:** 1 Institute of Parasitology, Biology Centre of the Czech Academy of Sciences Branišovská 31 370 05 České Budějovice Czech Republic; 2 Institute of Ecology, Nature Research Centre Akademijos 2 08412 Vilnius Lithuania; 3 Marine and Freshwater Research Centre, Galway-Mayo Institute of Technology H91 T8NW Galway Ireland; 4 BioSpecT EA7506, Faculty of Pharmacy, University of Reims Champagne-Ardenne 51 rue Cognacq-Jay 51096 Reims Cedex France; 5 Laboratory of Parasitology, Institute for Experimental Pathology, Keldur, University of Iceland IS-112 Reykjavík Iceland

**Keywords:** Trematoda, Morphology, Mitochondrial and nuclear DNA, Europe, North America, Mollusca

## Abstract

The biodiversity of freshwater ecosystems globally still leaves much to be discovered, not least in the trematode parasite fauna they support. Echinostome trematode parasites have complex, multiple-host life-cycles, often involving migratory bird definitive hosts, thus leading to widespread distributions. Here, we examined the echinostome diversity in freshwater ecosystems at high latitude locations in Iceland, Finland, Ireland and Alaska (USA). We report 14 echinostome species identified morphologically and molecularly from analyses of *nad1* and *28S rDNA* sequence data. We found echinostomes parasitising snails of 11 species from the families Lymnaeidae, Planorbidae, Physidae and Valvatidae. The number of echinostome species in different hosts did not vary greatly and ranged from one to three species. Of these 14 trematode species, we discovered four species (*Echinoparyphium* sp. 1, *Echinoparyphium* sp. 2, *Neopetasiger* sp. 5, and Echinostomatidae gen. sp.) as novel in Europe; we provide descriptions for the newly recorded species and those not previously associated with DNA sequences. Two species from Iceland (*Neopetasiger islandicus* and *Echinoparyphium* sp. 2) were recorded in both Iceland and North America. All species found in Ireland are new records for this country. Via an integrative taxonomic approach taken, both morphological and molecular data are provided for comparison with future studies to elucidate many of the unknown parasite life cycles and transmission routes. Our reports of species distributions spanning Europe and North America highlight the need for parasite biodiversity assessments across large geographical areas.

## Abstract (fr)

La biodiversité des écosystèmes d’eau douce à l’échelle mondiale laisse encore beaucoup à découvrir, notamment dans la faune parasitaire des trématodes qu’ils abritent. Les parasites trématodes Échinostomes ont des cycles de vie complexes à hôtes multiples impliquant souvent des oiseaux migrateurs comme hôtes définitifs, conduisant ainsi à des distributions étendues. Ici, nous avons examiné la diversité des échinostomes dans les écosystèmes d’eau douce à des latitudes élevées en Islande, Finlande, Irlande et en Alaska (États-Unis). Nous rapportons de séquences de *nad1* et d’ADNr *28S* morphologiquement et moléculairement à partir d’analyses de données de séquence d’ADNr *nad1* et *28S*. Nous avons trouvé des échinostomes parasitant les mollusques de 11 espèces des familles Lymnaeidae, Planorbidae, Physidae et Valvatidae. Le nombre d’espèces d’échinostomes dans différents hôtes ne variait pas beaucoup et allait d’une à trois espèces. Sur ces 14 espèces de trématodes, nous avons découvert quatre espèces (*Echinoparyphium* sp. 1, *Echinoparyphium* sp. 2, *Neopetasiger* sp. 5, Echinostomatidae gen. sp.) comme nouvelles pour l’Europe; nous fournissons des descriptions pour les espèces nouvellement signalées et celles qui n’étaient pas précédemment associées à des séquences d’ADN. Deux espèces d’Islande (*Neopetasiger islandicus* et *Echinoparyphium* sp. 2) ont été signalées en Islande et en Amérique du Nord. Toutes les espèces trouvées en Irlande sont de nouveaux signalements pour ce pays. Grâce à une approche taxonomique intégrative, des données morphologiques et moléculaires sont fournies à des fins de comparaison avec des études futures afin d’élucider les nombreux cycles de vie et voies de transmission des parasites, qui sont inconnus. Nos données sur la répartition des espèces en Europe et en Amérique du Nord soulignent la nécessité d’évaluer la biodiversité des parasites dans de vastes zones géographiques.

## Introduction

In recent years, it has been increasingly uncovered that trematodes commonly complete their life-cycles in freshwaters in the sub-Arctic, using the short summer seasons, with many hosts available at high densities [8, 31, 36, 66, 78, 122]. Particularly Iceland, with its nesting bird populations using the fertile lakes, is an area of worldwide importance for birds [65] and therefore for the trematode parasites associated with them. The Arctic freshwater ecosystems, used by trematodes, are consistently under pressure because of climate change, possibly leading to further biodiversity loss due to latitudinal range shifts of organisms from boreal regions [18, 59, 64, 112, 139]. However, maintaining and exploring freshwater biodiversity is crucial for understanding how various species contribute to the functioning of freshwater ecosystems [44, 139], and parasites are a fundamental part of this diversity [1, 117]. Trematodes from the family Echinostomatidae are influential players in freshwater ecosystems [81]. They can affect the larval trematode component community species composition in snail populations [83, 87], they contribute substantially to energy flow in ecosystems [11], and they can cause diseases in humans and wildlife [43, 130]. They are also considered effective competitors in combatting infection with schistosomes as the echinostome rediae actively feed on snail host tissue or other trematodes and can outcompete the less aggressive sporocysts [84]. Furthermore, a wide spectrum of first and second intermediate snail hosts to echinostomes has been revealed including multiple genera or even families of snails [24, 61, 84]. Because of this, echinostomes are frequently being used in ecological studies of host-parasite relationships [25], and they have been favoured in experimental model systems [39, 126].

The family Echinostomatidae Looss, 1899 is a fairly large group of trematodes with a cosmopolitan geographical distribution and with a remarkably diverse composition of genera (37 nominal genera recognised [126]), using a broad range of final vertebrate hosts (predominantly birds) [73]. Echinostomes, with a characteristic spined collar including a three-host life-cycle, have a wide range of second intermediate hosts (molluscs, other invertebrates, amphibians, and fishes) [69, 126]. Although they have received substantial attention from researchers and the family structure was recently re-evaluated based on the phylogenetic relationships of its members [126], their systematics are still non-satisfactory because of similar morphological characters between species, poor species diagnoses and convoluted synonymy [75]. There are several species complexes, particularly the “*Echinostoma revolutum*” group comprising species/lineages which have been defined as cryptic [24, 46, 48]. In recent years, in Europe and North America, but also in Africa, a substantial diversity of species of several genera was revealed via molecular genetic analyses; the species composition of the “*revolutum*” complex in Europe was partly solved, and new species representatives and species complexes (*Ec. trivolvis* and *Ec. robustum*) were recorded [24, 25, 32, 46, 48, 84, 118].

Particularly at the northern latitudes, including Iceland, Norway and northern Germany but also in Canada, new species were described and an unexpected diversity of echinostome trematodes was revealed in the associated freshwater ecosystems [51, 77, 118, 122], while in other regions (Alaska, Finland) data based only on morphology are available with molecular genetic data still lacking (see Table 1). These studies have pointed out the need for integrative taxonomy (with the preferred use of the mitochondrial gene *nad*1 as a marker) to further clarify a species status and to distinguish genetic lineages within species complexes [46]. Moreover, the first intermediate snail host species spectrum still remains to be explored as it turns out that with molecular data of both trematodes and snails different host spectra are revealed [78, 122].

**Table 1:** List of trematodes of the family Echinostomatidae recorded in freshwater in Iceland, Finland and Alaska, USA.

Species	Life-cycle stage	Host*	Reference
Iceland			
*Echinoparyphium recurvatum* (Linstow, 1873)	A	*Melanitta nigra* (L., 1758)	[14]
	C	*Radix peregra* (O.F. Müller, 1774)	[7]
*Echinostoma revolutum* (Frӧhlich, 1802)	A	*Clangula hyemalis* (L., 1758), *Melanitta nigra* (L., 1758)	[14]
	C	*Radix peregra* (O.F. Müller, 1774)	[48]
*Echinostoma* sp. IG	C	*Radix peregra* (O.F. Müller, 1774)	[48]
*Hypoderaeum conoideum* (Bloch, 1782)	M	*Radix peregra* (O.F. Müller, 1774)	[7]
*Neopetasiger islandicus* (Kostadinova & Skírnisson, 2007)	A	*Podiceps auritus* (L., 1758)	[77]
	C, M	*Gyraulus* cf. *laevis* (Alder, 1838), *Gasterosteus aculeatus* L., 1758	[47]
Finland			
*Echinoparyphium aconiatum* Dietz, 1909	C	*Lymnaea stagnalis* (L., 1758)	[76, 101, 132]
*Echinoparyphium recurvatum* (Linstow, 1873)	A	*Anas acuta* L., 1758, *A. crecca* L., 1758, *A. platyrhynchos* L., 1758, *Aythia fuligula* (L., 1758)	[13]
	C	*Lymnaea peregra* (O.F. Müller, 1774)	[76, 101, 132]
*Echinoparyphium* sp. 1	C	*Valvata macrostoma* Mӧrch, 1864	[37]
*Echinoparyphium* sp. 2	C	*Valvata macrostoma* Mӧrch, 1864	[37]
*Echinostoma revolutum* (Frӧhlich, 1802)	A	*Anas acuta* L., 1758, *A. clypeata* L., 1758, *A. crecca* L., 1758, *A. penelope* L., 1758, *A. platyrhynchos* L., 1758, *A. querquedula* L., 1758, *Aythya ferina* (L., 1758), *Ay. fuligula* (L., 1758)	[13]
	C	*L. stagnalis* (L., 1758), *L. peregra* (O.F. Müller, 1774)	[101, 132, 138]
*Hypoderaeum conoideum* (Bloch, 1782)	A	*Anas acuta* L., 1758, *A. crecca* L., 1758, *A. platyrhynchos* L., 1758,	[13]
	C	*L. stagnalis* (L., 1758)	[101, 132, 138]
Alaska, USA			
*Echinoparyphium aconiatum* Dietz, 1909	A	*Limnodromus scolopaceus* (Say, 1823)	[10]
*Echinoparyphium recurvatum* (Linstow, 1873)	A	*Larus hyperboreus* Gunnerus, 1767, *Limosa laponica* (L., 1758), *Pluvialis squatarola* (L., 1758)	[10, 16]
*Echinostoma calawayensis* Barker & Noll, 1915	A	*Ondatra zibethicus* (Linnaeus, 1766)	[70]
*Echinostoma trivolvis* (Cort, 1914)	A	*Calidris alpina* (L., 1758)	[17]

* Original names of hosts were used.

Our primary aim was to investigate the diversity of the echinostome trematode fauna in snails in freshwater lakes in Iceland. Since this oceanic island lies on the East Atlantic Flyway, it is an important nesting place, with high densities of aquatic birds visiting annually [22, 65, 82]. The trematode species so far discovered there were found to be non-endemic to Iceland. The three echinostome species recently found in Iceland include *Neopetasiger islandicus* Kostadinova & Skírnisson, 2007 afterwards also reported from North America [126], *Echinostoma revolutum* (Frölich, 1802) *sensu stricto* and *Echinostoma* sp. IG recorded from Europe [32, 46, 48]. Due to the apparent overlapping geographical distributions of echinostome species found in Iceland, we further included data from Ireland, Finland, and Alaska (USA) to investigate echinostome species diversity, host-use and distribution over a larger geographical area. We analysed novel DNA sequence data and associated them with morphological characterisations, together with data previously reported from Europe, North America, Africa, Asia and Australasia [24, 25, 46–48, 51, 84, 118, 122], with the aim of further contributing to the resolution of echinostome species diversity.

## Materials and methods

### Collection of material

A total of 6258 freshwater snails from Alaska, USA (May, June and July 2015), Finland (May–September 2007–2008), Iceland (June, July, August 2018–2019) and Ireland (July 2019) were collected for the present study (Table 2). The snails belonging to 11 species from the families Lymnaeidae, Planorbidae, Physidae and Valvatidae were collected in plastic containers with water from the locality and were brought to the laboratory. The snails were identified based on shell morphology using Burch [15] and Glöer [50]. In the laboratory, snails were placed into individual plastic cups filled with dechlorinated tap water and left for 24 h to detect natural emergence of cercariae. Emerged cercariae were examined live under a light microscope, Olympus BX51, BX41, photographed with the use of an attached digital camera and fixed in molecular grade ethanol for DNA isolation, and in 4% formalin solution for morphometric evaluation. Snails with emerging cercariae identified as belonging to the family Echinostomatidae were separated and the cercariae were subjected to subsequent molecular and morphological analyses. Thereafter, all snails were dissected under the dissecting stereomicroscope to detect all trematode intramolluscan stages (rediae). Vouchers of cercariae fixed in molecular grade ethanol and those fixed in formalin solution and transferred to 70% ethanol (see Table 3) are kept in the Helminthological collection of the Institute of Parasitology (IPCAS), Biology Centre of the Czech Academy of Sciences, České Budějovice, Czech Republic.

**Table 2:** Summary data on localities and snail species examined and infected with echinostomes.

Locality	Coordinates	Snail species	Sample size	No. infected	Prevalence (%)
Iceland					
Pond in Family Park, Reykjavík	64°08′14.5″ N, 21°52′02.6″ W	*Radix balthica*	352	4	1.1
Pond at Nordic House, Vatnsmýri Nature Reserve, Reykjavík	64°08′19″ N, 21°56′45″ W	*Radix balthica*	687	177	25.8
		*Physa acuta*	699	132	18.9
Lake Rauðavatn (near Reykjavík)	64°06′22.9″ N, 21°46′34.4″ W	*Radix balthica*	712	21	2.9
Lake Mývatn, Helgavogur	65°38′05.8″ N, 16°55′30.4″ W	*Radix balthica*	265	50	18.9
		*Gyraulus* cf. *parvus*	212	13	6.1
Lake Ashildarholtsvatn	65°44′00.6″ N, 19°37′23.8″ W	*Radix balthica*	244	24	9.8
		*Gyraulus* cf. *parvus*	206	9	4.4
Ireland					
Lough Corrib	53°21′27.0″ N, 9°04′36.0″ W	*Radix balthica*	573	10	1.7
		*Lymnaea stagnalis*	132	2	1.5
		*Myxas glutinosa*	60	1	1.7
		*Planorbarius corneus*	182	2	1.1
		*Planorbis planorbis*	32	2	6.3
Killeeneen	53°13′30.0″ N, 8°47′43.0″ W	*Lymnaea stagnalis*	10	1	10
Hackett Lough	53°29′27.0″ N 9°02′31.0″ W	*Lymnaea stagnalis*	39	1	2.6
Lough Mask	53°37′41.0″ N, 9°17′01.0″ W	*Radix balthica*	104	2	1.9
		*Stagnicola fuscus*	38	1	2.6
Alaska, USA					
Tanana, pool on river bank	64°15′26.4″ N, 146°09′46.6″ W	*Stagnicola elodes*	201	3	1.5
Fairbanks, small lake near airport	64°47′56.7″ N, 147°51′43.6″ W	*Radix auricularia*	1	1	100
Finland					
Lake Konnevesi	62°37′00.4″ N, 26°20′57.9″ E	*Valvata macrostoma*	1447	60	4.1
		*Myxas glutinosa*	18	1	5.6
		*Radix balthica*	27	1	3.7
Huumonjärvi	65°06′06.5″ N, 26°08′13.3″ E	*Lymnaea stagnalis*	17	9	52.9

**Table 3:** Summary data for the echinostomes and snail isolates used for generation of the *nad*1, *cox*1, *28S* and *ITS2* sequences in the present study.

Species	Isolate	Host species	Locality	GenBank ID		IPCAS No.
				*nad*1/*cox*1^a^	*28S*/*ITS2*^b^	
*Echinoparyphium aconiatum*	AF227	*Lymnaea stagnalis*	Ireland	MZ404641	MZ409801	D-825/E^c^
*Echinoparyphium aconiatum*	AF225	*Lymnaea stagnalis*	Ireland	MZ404642	–	–
*Echinoparyphium aconiatum*	AF226	*Lymnaea stagnalis*	Ireland	MZ404643	–	–
*Echinoparyphium aconiatum*	AF274	*Lymnaea stagnalis*	Finland	MZ404644	–	–
*Echinoparyphium aconiatum*	AF275	*Lymnaea stagnalis*	Finland	MZ404645	–	–
*Echinoparyphium aconiatum*	AF273	*Lymnaea stagnalis*	Finland	MZ404646	MZ409802	–
*Echinoparyphium recurvatum*	AF210	*Radix balthica*	Iceland	MZ404647	–	–
*Echinoparyphium recurvatum*	AF211	*Radix balthica*	Iceland	MZ404648	–	D-196/E
*Echinoparyphium recurvatum*	AF228	*Radix balthica*	Iceland	MZ404649	–	–
*Echinoparyphium recurvatum*	AF256	*Radix balthica*	Finland	MZ404650	–	–
*Echinoparyphium recurvatum*	AF229	*Radix balthica*	Ireland	MZ404651	–	–
*Echinoparyphium recurvatum*	AF222	*Radix balthica*	Ireland	MZ404652	–	–
*Echinoparyphium recurvatum*	AF205	*Radix balthica*	Iceland	MZ404653	–	–
*Echinoparyphium recurvatum*	AF220	*Radix balthica*	Iceland	MZ404654	–	D-196/F
*Echinoparyphium recurvatum*	AF254	*Myxas glutinosa*	Finland	MZ404655	MZ409803	–
*Echinoparyphium recurvatum*	AF255	*Myxas glutinosa*	Finland	MZ404656	–	–
*Echinoparyphium recurvatum*	AF204	*Radix balthica*	Iceland	MZ404657	MZ409804	–
*Echinoparyphium rubrum*	AF241	*Stagnicola elodes*	Alaska	MZ404658	MZ409805	D-833/E
*Echinoparyphium rubrum*	AF244	*Stagnicola elodes*	Alaska	MZ404659	MZ409806	D-833/F
*Echinoparyphium* sp. 1	AF251	*Valvata macrostoma*	Finland	MZ404660	–	D-834/E
*Echinoparyphium* sp. 1	AF252	*Valvata macrostoma*	Finland	MZ404661	MZ409807	–
*Echinoparyphium* sp. 1	AF253	*Valvata macrostoma*	Finland	MZ404662	–	D-834/F
*Echinoparyphium* sp. 2	AF421	*Physa acuta*	Iceland	MZ404663	–	D-835/E
*Echinoparyphium* sp. 2	AF420	*Physa acuta*	Iceland	MZ404664	MZ409808	–
*Echinoparyphium* sp. 2	AF423	*Physa acuta*	Iceland	MZ404665	–	D-835/F
*Echinostoma nasincovae*	AF232	*Planorbarius corneus*	Ireland	MZ404666	MZ409809	D-289/E
*Echinostoma revolutum s*. *str*.	AF206	*Radix balthica*	Iceland	MZ404667	MZ409810	D-130/E
*Echinostoma revolutum s*. *str*.	AF214	*Radix balthica*	Iceland	MZ404668	–	–
*Echinostoma revolutum s*. *str*.	AF219	*Radix balthica*	Iceland	MZ404669	–	–
*Echinostoma revolutum s*. *str*.	AF215	*Radix balthica*	Iceland	MZ404670	–	–
*Echinostoma revolutum s*. *str*.	AF216	*Radix balthica*	Iceland	MZ404671	–	–
*Echinostoma revolutum s*. *str*.	AF217	*Radix balthica*	Iceland	MZ404672	–	–
*Echinostoma revolutum*	AF235	*Radix auricularia*	Alaska	MZ404673	MZ409811	D-836/E/F
*Echinostoma revolutum*	AF236	*Stagnicola elodes*	Alaska	MZ404674	–	–
*Echinostoma revolutum*	AF237	*Stagnicola elodes*	Alaska	MZ404675	–	–
*Echinostoma* sp. IG	AF218	*Radix balthica*	Iceland	MZ404676	MZ409812	–
*Echinostoma* sp. IG	AF221	*Radix balthica*	Iceland	MZ404677	–	D-837/E
*Echinostoma* sp. IG	AF231	*Myxas glutinosa*	Ireland	MZ404678	MZ409813	–
*Hypoderaeum conoideum*	AF261	*Lymnaea stagnalis*	Finland	MZ404679	MZ409814	–
*Hypoderaeum conoideum*	AF262	*Lymnaea stagnalis*	Finland	MZ404680	–	–
*Hypoderaeum conoideum*	AF257	*Lymnaea stagnalis*	Finland	MZ404681	–	–
*Hypoderaeum conoideum*	AF259	*Lymnaea stagnalis*	Finland	MZ404682	–	D-138/E
*Moliniella anceps*	AF230	*Stagnicola fuscus*	Ireland	MZ404683	MZ409815	D-176/E
*Neopetasiger islandicus*	AF416	*Gyraulus* cf. *parvus*	Iceland	MZ404684	–	–
*Neopetasiger islandicus*	AF418	*Gyraulus* cf. *parvus*	Iceland	MZ404685	–	–
*Neopetasiger islandicus*	AF415	*Gyraulus* cf. *parvus*	Iceland	MZ404686	MZ409816	D-720/E
*Neopetasiger* sp. 5	AF233	*Planorbis planorbis*	Ireland	MZ404687	MZ409817	D-838/E
Echinostomatidae gen. sp.	AF258	*Lymnaea stagnalis*	Finland	MZ404688	MZ409818	–
Echinostomatidae gen. sp.	AF260	*Lymnaea stagnalis*	Finland	MZ404689	MZ409819	D-839/E/F
*Gyraulus* cf. *parvus*	AF351	–	Iceland	–	MZ400492^b^	–
*Gyraulus* cf. *parvus*	AF352	–	Iceland	MZ398103^a^	MZ400494^b^	–
*Gyraulus* cf. *parvus*	AF354	–	Iceland	MZ398105^a^	MZ400495^b^	–
*Gyraulus* cf. *parvus*	AF355	–	Iceland	–	MZ400491^b^	–
*Myxas glutinosa*	AF338	–	Ireland	MZ396110^a^	MZ400489^b^	–
*Physa acuta*	AF344	–	Iceland	MZ396244^a^	MZ400493^b^	–
*Radix balthica*	AF347	–	Iceland	–	MZ400490^b^	–
*Radix balthica*	AF348	–	Iceland	–	MZ400497^b^	–
*Radix balthica*	AF349	–	Iceland	–	MZ400496^b^	–
*Radix balthica*	AF353	–	Iceland	–	MZ400505^b^	–

a Sequence for cox1;

b sequence for ITS2;

c Abbreviations: E – molecular grade ethanol; F – fixed in formalin and transferred to 70% ethanol.

### DNA extraction, amplification, and sequencing

About 20–25 cercariae per sample were used for DNA extractions, following the protocol described by Georgieva et al. [48] (Table 3). Initially, to delineate and identify our isolates, we sequenced the section of the mitochondrial gene nicotinamide adenine dinucleotide dehydrogenase subunit 1 (*nad*1), following previous studies for echinostomes [24, 46, 48, 51, 84]. Thereafter, we sequenced the *28S* section of one or two isolates representing different species in our samples to compare them to species for which *nad*1 sequences were not available and identify the position of several species within the family Echinostomatidae. The section of the *nad*1 gene was amplified using the primers NDJ11 and NDJ2A [76, 97], following PCR conditions as described by Laidemitt et al. [84]. The section of the nuclear *28S rRNA* gene (*28S*) was amplified using primers digl2 and 1500R [121], following PCR conditions as described by Tkach et al. [126]. The amplified DNA was purified using exonuclease I and shrimp alkaline phosphatase enzymes [136] and sequenced using PCR primers and internal primers ECD2 and 300F [88, 89] for the *28S* section. Cycle sequencing of DNA was carried out applying ABI Big Dye™ v.3.1 chemistry at the commercial company SEQme (Dobříš, Czech Republic, https://www.seqme.eu) with the use of an AB3730x1 capillary sequencer. Sequences were assembled and edited using Geneious v. 11 (Biomatters, Auckland, New Zealand) and deposited in GenBank.

To confirm the morphology-based identification of snail species, the partial mitochondrial cytochrome *c* oxidase subunit 1 (*cox*1) gene and the internal transcribed spacer 2 (*ITS2*) sequences were generated for ten isolates (Table 3). The extraction protocol used was the same as for cercarial isolates (see above). The section of the *cox*1 gene was amplified using the primers LCO1490 and HC02198 and the protocol described by Folmer et al. [38] and the *ITS2* region was amplified using the primer pair RadITS2 and RADITS2RIXOR and the protocol described by Soldánová et al. [122].

### Phylogenetic analyses

Four alignments including novel and previously published sequences for echinostomes were built using MUSCLE [29] implemented in Geneious v. 11. Alignment 1 (431 nucleotides (nt)) included novel *nad*1 sequences of *Echinoparyphium* spp. (*n* = 25) and sequences of this genus available in GenBank (*n* = 31). The sequence of *Echinostoma revolutum* (KC618451) was used as the outgroup. Alignment 2 (417 nt) included *nad*1 sequences of *Echinostoma* spp. generated in this study (*n* = 13) and retrieved from GenBank (*n* = 27). The sequence of *Patagifer* sp. (MK534424) was used as the outgroup. Alignment 3 (402 nt) included *nad*1 sequences of *Neopetasiger* spp. generated in this study (*n* = 4) and retrieved from GenBank (*n* = 10). The sequence of *Drepanocephalus auritus* (KP053262) was used as the outgroup. The *nad*1 sequences were aligned with reference to the amino acid translation, using the trematode mitochondrial code (translation table 21) [42, 105]. Alignment 4 (1137 nt) included *28S* sequences obtained during the present study (*n* = 19) and sequences of other representatives of the family Echinostomatidae available in GenBank (*n* = 36). The sequence of *Caballerotrema* sp. (KT956941) was used as the outgroup. Taxa used as the outgroups were selected based on the results of the phylogenetic analyses of the Echinostomatoidea and *Echinostoma* published by Tkach et al. [126] and Georgieva et al. [46, 48].

Bayesian inference (BI) and maximum likelihood (ML) phylogenetic analyses were conducted using MrBayes version 3.2.3. [113] and PhyML version 3.0 [56] software, respectively. Prior to analyses, the best-fitting model was estimated with jModelTest 2.1.2 [23]. The general time-reversible model incorporating invariant sites and gamma distributed among-site rate variations (GTR + I + G) was selected for all datasets. Markov Chain Monte Carlo (MCMC) chains were run for 10,000,000 (Alignments 1, 2 and 4) or 3,000,000 (Alignment 3) generations, log-likelihood scores were plotted and only the final 75% of trees were used in BI analysis to produce the consensus trees. Nodal support for the ML analysis of all four alignments was estimated by performing 100 bootstrap pseudoreplicates. Trees were visualised using FigTree ver. 1.4 software [110]. Pairwise genetic distances were calculated using the p-distance model in MEGA ver. X [80]. New sequences of echinostomes were deposited in GenBank with accession numbers MZ404641–MZ404689 and MZ409801–MZ409819.

### Morphological evaluation

Cercariae and rediae were examined live under the light microscope Olympus BX51 and BX41 for primary identification based on their morphology, following the keys of Faltýnková et al. [34, 35]. Series of photomicrographs of live individuals and formalin fixed samples were taken with a digital camera on Olympus BX51 and BX41 microscopes; in locations with microscopes with no camera (Alaska, Finland), hand drawings of live cercariae were made. Measurements for each isolate were taken from the digital images with the aid of QuickPHOTO CAMERA 2.3 image analysis software. Metrical data in the descriptions are based on live specimens and fixed material (formalin and/or ethanol). All measurements in the descriptions are in micrometres and are presented as the range, followed by the mean in parentheses. Measurements of fixed material are provided separately.

## Results

In total, 14 echinostome species were identified infecting snails from four different families: Lymnaeidae, Planorbidae, Physidae and Valvatidae, sampled in Alaska, Iceland, Finland and Ireland.

### DNA-based identification

**Figure 1:**
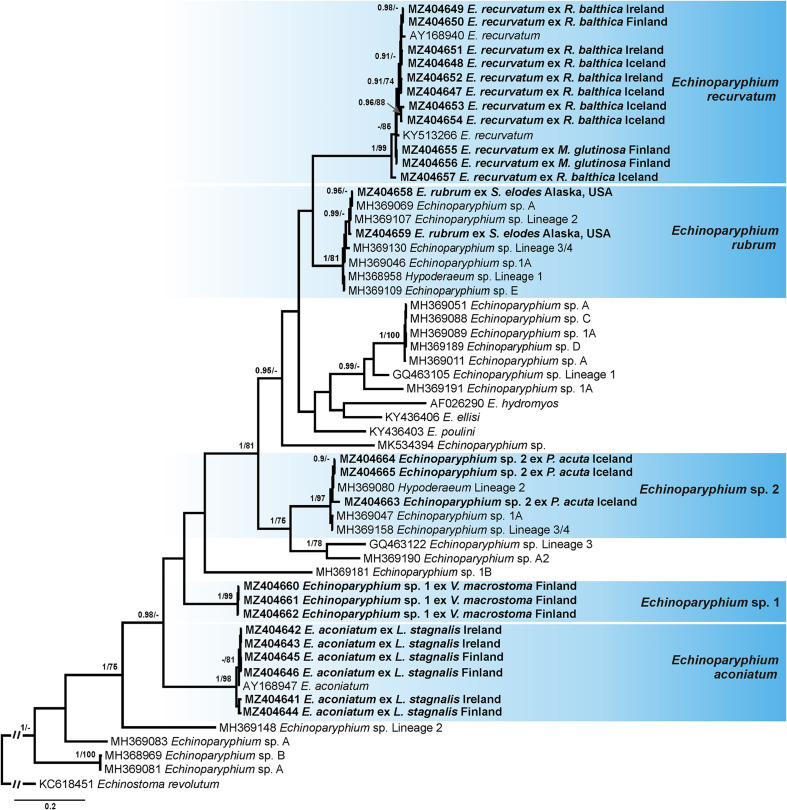
Phylogram from Bayesian inference (BI) analysis based on the *nad*1 sequences of *Echinoparyphium* spp. Nodal support values are given as BI/ML (maximum likelihood). Support values lower than 0.90 (BI) and 70 (ML) are not shown. The scale-bar indicates the expected number of substitutions per site. Newly generated sequences are highlighted in bold. Coloured rectangles indicate species identified in this study.

**Figure 2:**
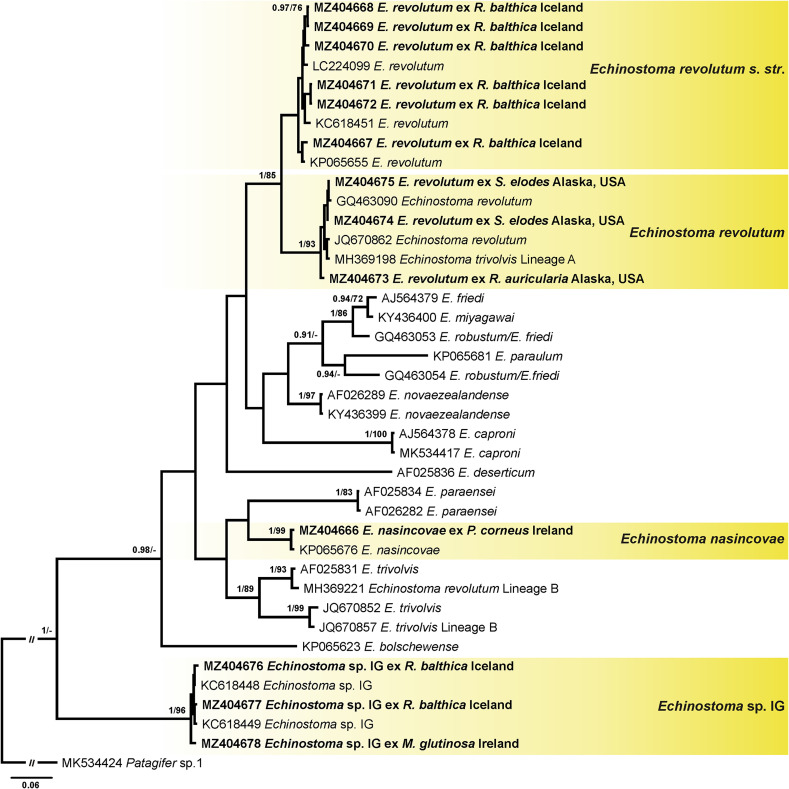
Phylogram from Bayesian inference (BI) analysis based on the *nad*1 sequences of *Echinostoma* spp. Nodal support values are given as BI/ML (maximum likelihood). Support values lower than 0.90 (BI) and 70 (ML) are not shown. The scale-bar indicates the expected number of substitutions per site. Newly generated sequences are highlighted in bold. Coloured rectangles indicate species identified in this study.

**Figure 3:**
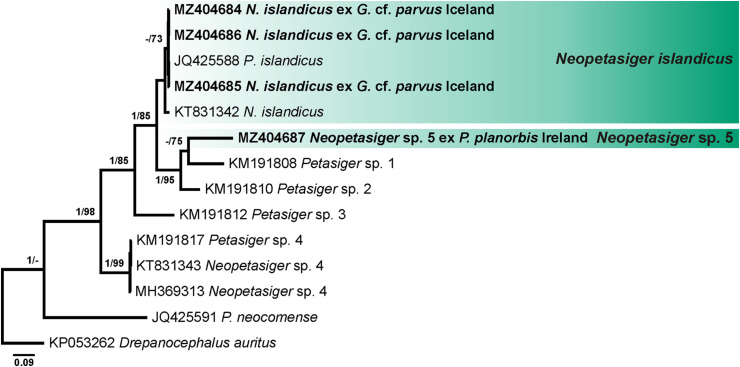
Phylogram from Bayesian inference (BI) analysis based on the *nad*1 sequences of *Neopetasiger* spp. Nodal support values are given as BI/ML (maximum likelihood). Support values lower than 0.90 (BI) and 70 (ML) are not shown. The scale-bar indicates the expected number of substitutions per site. Newly generated sequences are highlighted in bold. Coloured rectangles indicate species identified in this study.

**Figure 4:**
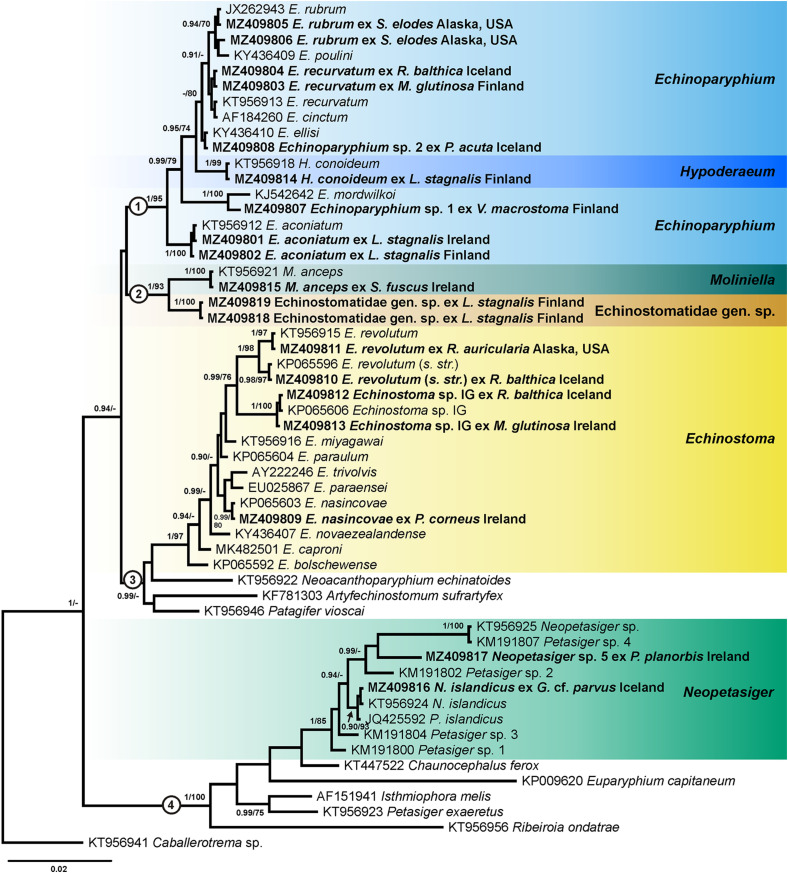
Phylogram from Bayesian inference (BI) analysis based on the *28S* sequences of the representatives of the Echinostomatidae. Nodal support values are given as BI/ML (maximum likelihood). Support values lower than 0.90 (BI) and 70 (ML) are not shown. The scale-bar indicates the expected number of substitutions per site. Newly generated sequences are highlighted in bold. Coloured rectangles indicate species identified in this study. The numbers 1, 2, 3 and 4 refer to the clades discussed in the text.

During the present study, 68 novel sequences, including 19 of *28S rDNA* and 49 of *nad*1 were generated for 49 cercarial isolates belonging to the family Echinostomatidae (Table 3). Molecular delineation of the isolates and their species identification was performed based on the analysis of *nad*1 sequence data via comparison to previously published data for echinostomes (Table 4). Analyses of the *28S rDNA* sequence data were conducted to explore relationships among collected taxa and to identify several species for which *nad*1 sequences were not available in GenBank. Cercariae of *Echinoparyphium rubrum* (Cort, 1917) and *Moliniella anceps* (Molin, 1859) were molecularly identified based on the *28S* sequence data analyses. For *Echinostoma revolutum* we follow the concept of Georgieva et al. [46] and use *Ec. revolutum sensu stricto* (*s. str.*) for European isolates and *Ec. revolutum* of Detwiler et al. [24] for North American isolates. An unknown species of *Neopetasiger* obtained in the present study was named using the subsequent number following the study of Selbach et al. [118]. Pairwise genetic distances of the highlighted clades (see Figs. 1–4) are presented in the Supplementary Tables S1, S2, S3 and S4.

**Table 4:** List of trematodes of the superfamily Echinostomatoidea used in the phylogenetic analyses.

Species	Host species*	Locality	GenBank ID, *nad*1	GenBank ID, *28S*	Reference
*Artyfechinostomum sufrartyfex*	*Sus scrofa domestica*	India	–	KF781303	Tandon et al. (unpublished)
*Caballerotrema* sp.	*Arapaima gigas*	Peru	–	KT956941	[126]
*Chaunocephalus ferox*	*Ciconia nigra*	Ukraine	–	KT447522	[55]
*Drepanocephalus auritus*	*Planorbella trivolvis*	USA	KP053262	–	[108]
*Echinoparyphium aconiatum*	*Lymnaea stagnalis*	Finland	AY168947	–	[76]
*Echinoparyphium aconiatum*	*Lymnaea stagnalis*	Czech Republic	–	KT956912	[126]
*Echinoparyphium cinctum*	*Anas platyrhynchos*	Ukraine	–	AF184260	[128]
*Echinoparyphium ellisi*	*Anas platyrhynchos*	New Zealand	KY436406	–	[45]
*Echinoparyphium ellisi*	*Anas platyrhynchos*	New Zealand	–	KY436410	[45]
*Echinoparyphium hydromyos*	*Hydromys chrysogaster*	Australia	AF026290	–	[97]
*Echinoparyphium mordwikoi*	*Valvata piscinalis*	Lithuania	–	KJ542642	[123]
*Echinoparyphium recurvatum*	*Lymnaea peregra*	UK	AY168940	–	[76]
*Echinoparyphium recurvatum*	*Sphaerium* sp.	Norway	KY513266	–	[122]
*Echinoparyphium recurvatum*	*Radix ovata*	Slovakia	–	KT956913	[126]
*Echinoparyphium rubrum*	*Helisoma trivolvis*	USA	–	JX262943	[129]
*Echinoparyphium* sp. A	Not specified	Canada	MH369069	–	[51]
*Echinoparyphium* sp. Lineage 2	Not specified	Canada	MH369107	–	[51]
*Echinoparyphium* sp. Lineage 3	*Helisoma trivolvis*	USA	GQ463122	–	[24]
*Echinoparyphium* sp. Lineage 3/4	*Helisoma trivolvis*	Canada	MH369158	–	[51]
Echinoparyphium sp. 1A	Not specified	Canada	MH369046	–	[51]
*Echinoparyphium* sp. 1A	Not specified	Canada	MH369047	–	[51]
*Echinoparyphium* sp. A2	*Physa gyrina*	Canada	MH369190	–	[51]
*Echinoparyphium* sp. Lineage 2	Not specified	Canada	MH369148	–	[51]
*Echinoparyphium* sp. A	Not specified	Canada	MH369081	–	[51]
*Echinoparyphium* sp. A	Not specified	Canada	MH369083	–	[51]
*Echinoparyphium* sp. A	Not specified	Canada	MH369051	–	[51]
*Echinoparyphium* sp. A	Not specified	Canada	MH369011	–	[51]
*Echinoparyphium* sp. B	*Stagnicola elodes*	Canada	MH368969	–	[51]
*Echinoparyphium* sp. C	*Stagnicola elodes*	Canada	MH369088	–	[51]
*Echinoparyphium* sp. D	*Stagnicola elodes*	Canada	MH369189	–	[51]
*Echinoparyphium* sp. E	Not specified	Canada	MH369109	–	[51]
*Echinoparyphium* sp. 1A	Not specified	Canada	MH369089	–	[51]
*Echinoparyphium* sp. 1A	Not specified	Canada	MH369191	–	[51]
*Echinoparyphium* sp. 1B/A2	Not specified	Canada	MH369181	–	[51]
*Echinoparyphium* sp. Lineage 1	*Ondatra zibethicus*	USA	GQ463105	–	[24]
*Echinoparyphium* sp. Lineage 3/4	*Helisoma trivolvis*	Canada	MH369130	–	[51]
*Echinoparyphium* sp.	*Bulinus tropicus*	Kenya	MK534394	–	[84]
*Echinoparyphium poulini*	*Cygnus atratus*	New Zealand	KY436403	–	[45]
*Echinoparyphium poulini*	*Cygnus atratus*	New Zealand	–	KY436409	[45]
*Echinostoma bolschewense*	*Viviparus acerosus*	Slovakia	–	KP065592	[46]
*Echinostoma bolschewense*	*Viviparus acerosus*	Slovakia	KP065623	–	[46]
*Echinostoma caproni*	*Rattus norvegicus*	Egypt	AJ564378	–	Marcilla et al. (unpublished)
*Echinostoma caproni*	*Biomphalaria sudanica*	Kenya	MK534417	–	[84]
*Echinostoma caproni*	*Biomphalaria sudanica*	Kenya	–	MK482501	[84]
*Echinostoma deserticum*	–	Niger	AF025836	–	[97]
*Echinostoma friedi*	*Mesocricetus auratus*	Spain	AJ564379	–	Marcilla et al. (unpublished)
*Echinostoma* IG	*Radix peregra*	Iceland	KC618448	–	[48]
*Echinostoma* IG	*Radix auricularia*	Germany	KC618449	–	[48]
*Echinostoma* IG	*Radix auricularia*	Germany	–	KP065606	[46]
*Echinostoma miyagawai*	*Anas platyrhynchos*	New Zealand	KY436400	–	[45]
*Echinostoma miyagawai*	*Anas platyrhynchos*	Ukraine	–	KT956916	[126]
*Echinostoma nasincovae*	*Planorbarius corneus*	Czech Republic	KP065676	–	[46]
*Echinostoma nasincovae*	*Planorbarius corneus*	Czech Republic	–	KP065603	[46]
*Echinostoma novaezealandense*	*Branda canadensis*	New Zealand	AF026289	–	[97]
*Echinostoma novaezealandense*	*Anas platyrhynchos*	New Zealand	KY436399	–	[45]
*Echinostoma novaezealandense*	*Cygnus atratus*	New Zealand	–	KY436407	[45]
*Echinostoma paraensei*	–	Brazil	AF025834	–	[97]
*Echinostoma paraensei*	*Glyptophysa* sp.	Australia	AF026282	–	[97]
*Echinostoma paraensei*	“hamster”	USA	–	EU025867	Brant et al. (unpublished)
*Echinostoma paraulum*	*Lymnaea stagnalis*	Germany	KP065681	–	[46]
*Echinostoma paraulum*	*Lymnaea stagnalis*	Germany	–	KP065604	[46]
*Echinostoma revolutum*	*Lymnaea elodes*	USA	GQ463090	–	[24]
*Echinostoma revolutum*	*Ondatra zibethicus*	USA	JQ670862	–	[25]
*Echinostoma revolutum*	*Aythya collaris*	USA	–	KT956915	[126]
*Echinostoma revolutum s*. *str*.	*Anas platyrhynchos*	Bangladesh	LC224099	–	[96]
*Echinostoma revolutum s*. *str*.	*Radix peregra*	Iceland	KC618451	–	[48]
*Echinostoma revolutum s*. *str*.	*Lymnaea stagnalis*	Finland	KP065655	–	[46]
*Echinostoma revolutum s*. *str*.	*Aythya fuligula*	Czech Republic	–	KP065596	[46]
*Echinostoma revolutum* Lineage B	*Stagnicola elodes*	Canada	MH369221	–	[51]
*Echinostoma robustum/E. friedi*	*Lymnaea elodes*	USA	GQ463053	–	[24]
*Echinostoma robustum/E. friedi*	*Lymnaea elodes*	USA	GQ463054	–	[24]
*Echinostoma trivolvis*	–	North America	AF025831	–	[97]
*Echinostoma trivolvis*	*Mesocricetus auratus*	UK	–	AY222246	[106]
*Echinostoma trivolvis*	*Ondatra zibethicus*	USA	JQ670852	–	[25]
*Echinostoma trivolvis* Lineage A	*Helisoma trivolvis*	Canada	MH369198	–	[51]
*Echinostoma trivolvis* Lineage B	*Ondatra zibethicus*	USA	JQ670857	–	[25]
*Euparyphium capitaneum*	*Anhinga anhinga*	USA	–	KP009620	[79]
*Hypoderaeum conoideum*	*Anas platyrhynchos*	Ukraine	–	KT956918	[126]
*Hypoderaeum* Lineage 1	*Stagnicola elodes*	Canada	MH368958	–	[51]
*Hypoderaeum* Lineage 1/2	*Stagnicola elodes*	Canada	MH369080	–	[51]
*Isthmiophora melis*	*Nyctereutes procyonoides*	Ukraine	–	AF151941	[127]
*Moliniella anceps*	*Planorbarius corneus*	Lithuania	–	KT956921	[126]
*Neoacanthoparyphium echinatoides*	*Viviparus acerosus*	Slovakia	–	KT956922	[126]
*Neopetasiger islandicus*	*Planorbula armigera*	Canada	KT831342	–	[52]
*Neopetasiger islandicus*	*Aechmophorus occidentalis*	USA	–	KT956924	[126]
*Neopetasiger neocomense*	*Podiceps cristatus*	Czech Republic	JQ425591	–	[47]
*Neopetasiger* sp.	*Podiceps grisegena*	USA	–	KT956925	[126]
*Neopetasiger* sp. 4	*Helisoma trivolvis*	Canada	KT831343	–	[52]
*Neopetasiger* sp. 4	*Helisoma trivolvis*	Canada	MH369313	–	[51]
*Patagifer vioscai*	*Eudocimus albus*	USA	–	KT956946	[126]
*Patagifer* sp. 1	*Biomphalaria sudanica*	Kenya	MK534424	–	[84]
*Petasiger islandicus*	*Gyraulus* cf. *laevis*	Iceland	JQ425588	–	[47]
*Petasiger islandicus*	*Gyraulus* cf. *laevis*	Iceland	–	JQ425592	[47]
*Petasiger exaeretus*	*Phalacrocorax carbo*	Ukraine	–	KT956923	[126]
*Petasiger* sp. 1	*Gyraulus albus*	Germany	KM191808	–	[118]
*Petasiger* sp. 1	*Planorbis planorbis*	Czech Republic	–	KM191800	[118]
*Petasiger* sp. 2	*Gyraulus albus*	Germany	KM191810	–	[118]
*Petasiger* sp. 2	*Gyraulus albus*	Germany	–	KM191802	[118]
*Petasiger* sp. 3	*Gyraulus albus*	Germany	KM191812	–	[118]
*Petasiger* sp. 3	*Planorbis planorbis*	Germany	–	KM191804	[118]
*Petasiger* sp. 4	*Gasterosteus aculeatus*	Canada	KM191817	–	[118]
*Petasiger* sp. 4	*Gasterosteus aculeatus*	Canada	–	KM191807	[118]
*Ribeiroia ondatrae*	*Pelecanus erythrorhynchos*	USA	–	KT956956	[126]

* Host names are used as in publications.

The newly generated *nad*1 sequences for isolates of *Echinoparyphium* spp. clustered in five strongly supported clades (Fig. 1) in the tree resulting from BI and ML analyses of the first alignment: five isolates collected from *Radix balthica* (Linnaeus) in Iceland, three isolates from *R*. *balthica* in Ireland and three isolates from *R*. *balthica* and *M*. *glutinosa* (O.F. Müller) in Finland clustered with isolates of *E*. *recurvatum* (Linstow, 1873) previously reported in Europe [76, 122]; two isolates collected from *Stagnicola elodes* (Say) in Alaska which were identified as *E*. *rubrum* clustered with five isolates of unidentified species of *Echinoparyphium* (five species) and one isolate of unidentified species of *Hypoderaeum* reported in Canada [51]; three isolates collected from *Physa acuta* Draparnaud in Iceland, to which we refer as *Echinoparyphium* sp. 2, clustered with two isolates of unidentified species of *Echinoparyphium* and one isolate of unidentified species of *Hypoderaeum* reported in Canada [51]; three isolates collected from *Valvata macrostoma* Mörch in Finland, to which we refer as *Echinoparyphium* sp. 1, formed a separate clade to other *Echinoparyphium* spp.; and three isolates from *Lymnaea stagnalis* (Linnaeus) collected in Ireland and three isolates from *L*. *stagnalis* in Finland clustered with an isolate of *E*. *aconiatum* Dietz, 1909 previously reported in Europe [76]. The sequence divergence between isolates in clade “*E*. *aconiatum*” was 0–1.4% (0–5 nt), between isolates in clade “*E*. *recurvatum*” it was 0–3.4% (0–12 nt), between isolates in clade “*E*. *rubrum*” it was 0–1.9% (0–8 nt), and between isolates in clade “*Echinoparyphium* sp. 2” it was 0–2.4% (0–8 nt) (Alignment 1; 417 nt). Sequences of *Echinoparyphium* sp. 1 were identical.

The phylogenetic tree resulting from BI and ML analyses of the data in the second alignment showed that newly generated *nad*1 sequences of isolates of *Echinostoma* spp. clustered within four strongly supported clades corresponding to four species (Fig. 2): six isolates collected from *R*. *balthica* in Iceland clustered with isolates of *Ec*. *revolutum* (Frölich, 1802) *s*. *str*. previously reported in Europe including Iceland [46, 48]; three isolates collected from *Radix auricularia* (Linnaeus) and *S*. *elodes* in Alaska clustered with *Ec*. *revolutum* previously reported in the USA [24, 25] and *Ec*. *trivolvis* Lineage A reported from Canada [51]; one isolate collected from *Planorbarius corneus* (Linnaeus) in Ireland clustered with the species *Echinostoma nasincovae* Faltýnková, Georgieva, Soldánová & Kostadinova, 2015 recently described in Europe [32]; and two isolates collected from *R*. *balthica* in Iceland and one isolate from *M. glutinosa* in Ireland clustered with an unidentified species of *Echinostoma* sp. IG *sensu* Georgieva et al. [48] previously reported in Iceland, Germany and Wales (UK) [48]. The sequence divergence between isolates of *Ec*. *nasincovae* was 0.7% (3 nt), between isolates of *Ec*. *revolutum s*. *str*. it was 0–1.6% (0–7 nt), between isolates of *Ec*. *revolutum* it was 0–0.9% (0–4 nt) and between isolates of *Echinostoma* sp. IG it was 0.2–1.2% (1–5 nt) (Alignment 2; 430 nt).

Both BI and ML analyses based on *nad*1 sequences of *Neopetasiger* spp. in our third alignment resulted in consensus trees with similar topologies (Fig. 3). Four isolates of *Neopetasiger* collected in the present study in *Gyraulus* cf. *parvus* and *Planorbis planorbis* (Linnaeus) in Iceland and Ireland, respectively fall into two strongly supported clades (Fig. 3). Three identical isolates representing species of *N*. *islandicus* Kostadinova & Skírnisson, 2007 clustered with two isolates of the same species from *G*. cf. *laevis* and *Planorbula armigera* (Say) in Iceland and Canada, respectively (Fig. 3). The intraspecific divergence between the four European isolates of this species was 0–0.3% (0–1 nt) (Alignment 3; 402 nt). Isolates from North America differed from the European isolates by 2.8–3.1% (10–11 nt). The remaining isolate collected in Ireland formed a separate branch within the clade consisting of *Neopetasiger* sp. 1 and *Neopetasiger* sp. 2 previously reported from *G*. *albus* in Germany [118]. The interspecific divergence between sequences of *Neopetasiger* sp. 5 and the other species of this genus included in the analyses was 18.5–32.5% (66–116 nt). *Neopetasiger* sp. 2 appeared to be more closely related to *Neopetasiger* sp. 5, whereas *N*. *neocomense* showed the highest sequence divergence.

Comparison of *nad*1 sequence data between the isolate of *Hypoderaeum conoideum* (Bloch, 1782) of the present study and two isolates of this species available in GenBank (AY168949 [76]; and MH282580 [95]) showed low divergence (0.2–0.5%,1–2 nt), confirming identification of our isolate as *H. conoideum*.

The results of phylogenetic analyses based on *28S rDNA* sequences (fourth alignment) confirmed the species delineation and identification based on *nad*1 data analyses. The novel sequences clustered within the four clades presented (Fig. 4). Clade 1 included sequences of *Echinoparyphium* spp. and *H*. *conoideum*. Our sequences of *E*. *aconiatum*, *E*. *recurvatum*, *E*. *rubrum* and *H*. *conoideum* clustered with sequences of the same species retrieved from GenBank. The isolate of *Echinoparyphium* sp. 1 clustered with the isolate of *E*. *mordwilkoi* Skrjabin, 1915 with strong support (1/100), while the sequence divergence between these isolates was 0.6% (7 nt) demonstrating that they represent different species. The isolate of *Echinoparyphium* sp. 2 clustered with isolates of *E*. *ellisi* (Johnston & Simpson, 1944), and the sequences of these isolates were identical.

Clade 2 consisted of two isolates of *M*. *anceps* and two isolates of an unidentified species Echinostomatidae gen. sp. The isolate of *M*. *anceps* in our study was collected from the snail *Stagnicola fuscus* (C. Pfeiffer) in Ireland and two isolates of Echinostomatidae gen. sp. were collected from *L*. *stagnalis* in Finland. The sequence divergence between the two species within the *28S rDNA* dataset was 1.2% (13 nt) and within the *nad*1 dataset it was 22.5% (97 nt).

Clade 3 included sequences of *Echinostoma* and sequences of *Neoacanthoparyphium*, *Artyfechinostomum* and *Patagifer* at basal position. Our sequences of *Ec*. *nasincovae*, *Ec*. *revolutum s*. *str*., *Ec*. *revolutum*, and *Echinostoma* sp. IG clustered into a strongly supported clade (1/97) with the sequences of corresponding species obtained from GenBank.

Within Clade 4, our isolates of *N*. *islandicus* and *Neopetasiger* sp. 5 clustered in a strongly supported subclade (1/85) with isolates representing members of the genus *Neopetasiger*.

Based on the results of molecular identification, our samples represented 14 species belonging to six genera: *Echinoparyphium* (*E*. *aconiatum*, *E*. *recurvatum*, *E*. *rubrum*, *Echinoparyphium* sp. 1 and *Echinoparyphium* sp. 2), *Echinostoma* (*Ec*. *nasincovae*, *Ec*. *revolutum s*. *str*., *Ec*. *revolutum* and *Echinostoma* sp. IG), *Neopetasiger* (*N*. *islandicus* and *Neopetasiger* sp. 5), *Hypoderaeum* (*H. conoideum*), *Moliniella* (*M. anceps*) and one unidentified species Echinostomatidae gen. sp.

The incorporation of the *nad*1 sequences of echinostomes (*Echinoparyphium*, *Echinostoma* and *Hypoderaeum*) published by Gordy and Hanington [51] in our analyses demonstrated numerous incorrect taxonomic annotations. In particular, (i) different names were used for the same species (Fig. 1, clades of “*E. rubrum*”, “*Echinoparyphium* sp. 2” and clade with *Echinoparyphium* sp. A, 1A, C, D, Lineage A); (ii) the same name was used for different species (Fig. 1, *Echinoparyphium* sp. 1A (MH369046 and MH369047), and *Echinoparyphium* sp. A (MH369069, MH369051, MH369083 and MH369081)); and (iii) different species names for sequences have the same GenBank accession number (i.e., MH369130 and MH369158 correspond to *Echinoparyphium* sp. Lineage 3 and *Echinoparyphium* sp. Lineage 4; MH369080 corresponds to *Hypoderaeum* sp. Lineage 1 and *Hypoderaeum* sp. Lineage 2). Our analyses demonstrated that the genus of cercariae reported as *Hypoderaeum* sp. Lineages 1 and 2 (MH368958 and MH369080) was misidentified. These cercariae belong to the species of *Echinoparyphium* (Fig. 1). Additionally, the previous identifications of *Echinostoma* spp. were not followed (Fig. 2, see clades of “*Ec*. *revolutum*” and “*Ec*. *trivolvis*”). Another problem was related to the data of the host range of echinostomes. Although the species of snail hosts were provided in the paper, these names were not associated with the specific isolates, which precludes identification of the host of each species considering that some of the isolates were misidentified.

A total of 14 *cox*1 (*n* = 4) and *ITS2* (*n* = 10) sequences were generated for snail isolates (Table 3). Molecular identification was achieved via comparison of novel sequences with those previously published and available in GenBank. Generally, the sequence divergence was low corresponding to the intraspecific level. The *cox*1 sequence of *P*. *acuta* from Iceland differed from *P*. *acuta* from Greece (KF737936; [4]) and the USA (KJ769124; [57]) by 0.2–1.4% (1–9 nt). The *ITS2* sequence of *P*. *acuta* from Iceland differed from *P*. *acuta* from Mexico (HQ283272; [20]) and the USA (KF316326, KF316328; [102]) by 1–2.1% (3–6 nt). The *cox*1 sequence of our isolate *M*. *glutinosa* collected in Ireland differed from *M*. *glutinosa* in the USA (EU818798; [3]) and in Europe (DQ980191; [107]) by 0.3% (1 nt). The *ITS2* sequence of our isolate and a sequence of *M*. *glutinosa* from the UK (MN644819; [114]) were identical. The identification of *R*. *balthica* is described in Kudlai et al. [78]. Four additional sequences of *R*. *balthica* from Iceland were obtained in this study. The intraspecific divergence was 0–0.2% (0–1 nt). These sequences were compared to the *ITS2* sequence from Iceland (HQ003227–HQ003229; [66]), Norway (KY513276–KY513278; [122]), UK (KT337593, KT337601; [85]), Germany (HE573078; [116]), Switzerland (HE573081; [116]) and Spain (HE573099; [116]). The sequence divergence was low (0–0.7% (2 nt)), corresponding to the intraspecific level.

The intraspecific difference between *cox*1 sequences of *Gyraulus* cf. *parvus* generated in our study was 0.4% (2 nt). They differed from sequences of *Gyraulus parvus* from the USA (LC429535; [115]) and from Canada (MG421286, MG421343, MG421564; [134]) by 4–6.1% (22–33 nt). The *ITS2* sequences of *Gyraulus* cf. *parvus* generated in the present study were identical and differed from a sequence of *G*. *parvus* in GenBank from Canada (MN644828; [114]) by 0.5% (3 nt).

### Morphological characterisation

The morphology of all the present cercariae corresponds well to that of the family Echinostomatidae Looss, 1899 in the presence of a head collar with a row of collar spines larger than tegumental spines, the main ascending excretory channels filled with refractive granules, and a simple tail with or without fin-folds [34, 35, 41, 62, 73]. Species and genera are listed alphabetically. Descriptions are provided only for newly recorded species (*Echinoparyphium* sp. 1, *Ec. revolutum*, *Neopetasiger* sp. 5 and Echinostomatidae gen. sp.) and those not previously associated with DNA sequences (*E. recurvatum*, *E. rubrum* and *Echinoparyphium* sp. 2).

#### Echinostomatidae Looss, 1899

##### *Echinoparyphium* Dietz, 1909

###### *Echinoparyphium aconiatum* Dietz, 1909

*First intermediate host*: *Lymnaea stagnalis* (Linnaeus) (Gastropoda: Lymnaeidae).

*Localities*: Hackett Pond, Killeeneen, Lough Corrib, Ireland; Huumonjärvi, Finland.

*Representative DNA sequences*: MZ404641–MZ404646 (*nad*1); MZ409801, MZ409802 (*28S*).

*Remarks*: The new material from Ireland and Finland keys down to *E*. *aconiatum* in the key of Faltýnková et al. [34]. *Echinoparyphium aconiatum* is a parasite of anatid birds (ducks, geese) in the Holarctic [119]; in Europe, its larval stages are among those most commonly found in *L. stagnalis* [33, 34]. This is the first species record for Ireland and the species’ most western distribution in Europe.

###### *Echinoparyphium recurvatum* (Linstow, 1873) Dietz, 1909

*First intermediate hosts*: *Radix balthica* (Linnaeus), *Myxas glutinosa* (O.F. Müller) (Gastropoda: Lymnaeidae).

*Localities*: Lake Ashildarholtsvatn, Lake Rauðavatn, pond at Nordic House, Iceland; Lough Corrib, Lough Mask, Ireland; Lake Konnevesi, Finland.

*Representative DNA sequences*: MZ404647–MZ404657 (*nad*1); MZ409803, MZ409804 (*28S*).

**Figure 5:**
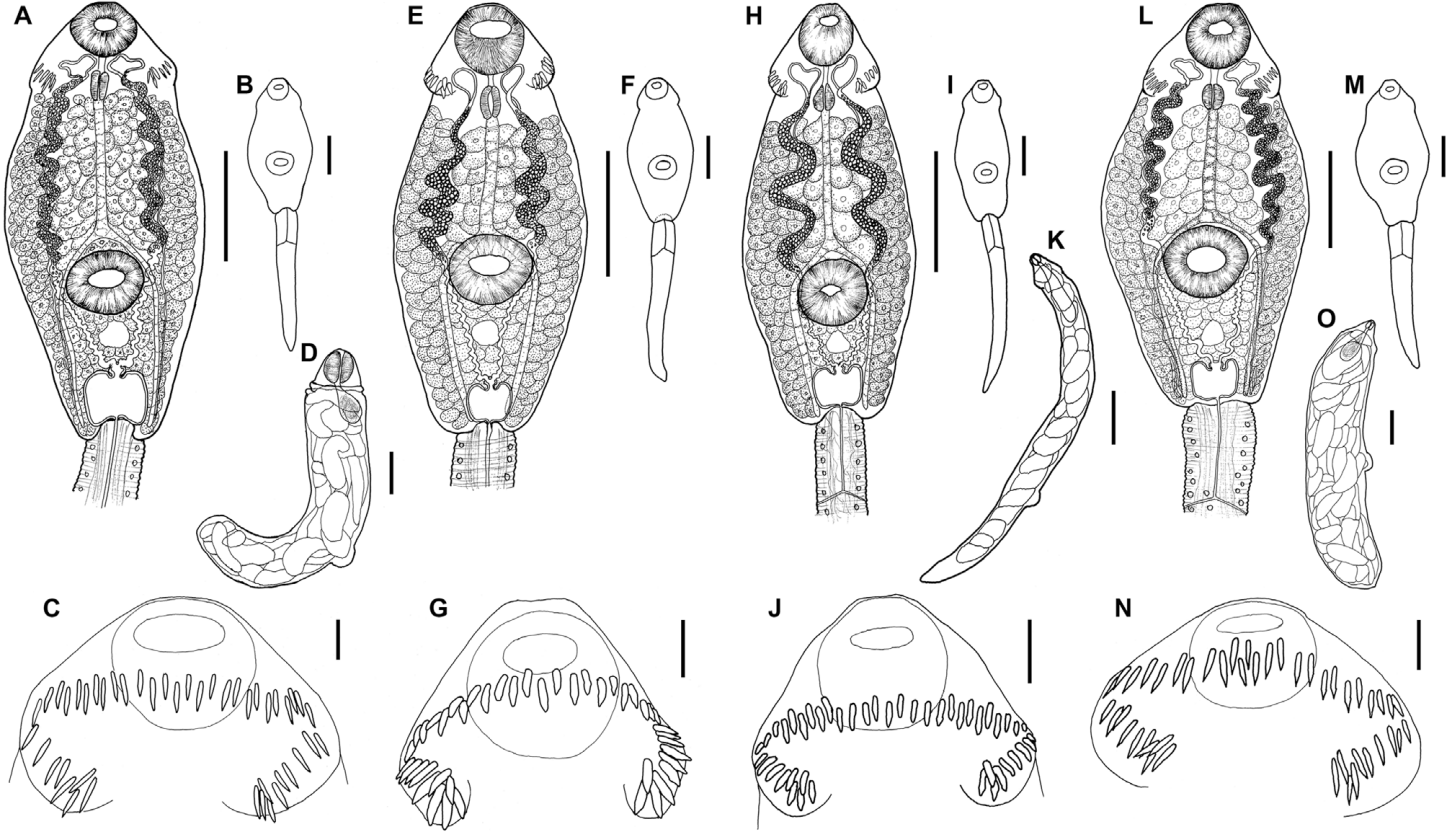
Drawings of cercariae. *Echinoparyphium recurvatum* ex *Radix balthica*. (A) body, ventral view, (B) total view, (C) head collar with collar spines, (D) redia, lateral view. *Echinoparyphium rubrum* ex *Stagnicola elodes*. (E) body, ventral view, (F) total view, (G) head collar with collar spines. *Echinoparyphium* sp. 1 ex *Valvata macrostoma*. (H) body, ventral view, (I) total view, (J) head collar with collar spines, (K) redia, lateral view. *Echinoparyphium* sp. 2 ex *Physa acuta*. (L) body, ventral view, (M) total view, (N) head collar with collar spines, (O) redia, lateral view. Scale-bars: A, B, E, F, H, L, M, 100 μm; C, G, J, N, 20 μm; D, I, K, O, 200 μm.

Cercaria (Figs. 5A–5C, 7A–7C)

**Figure 6:**
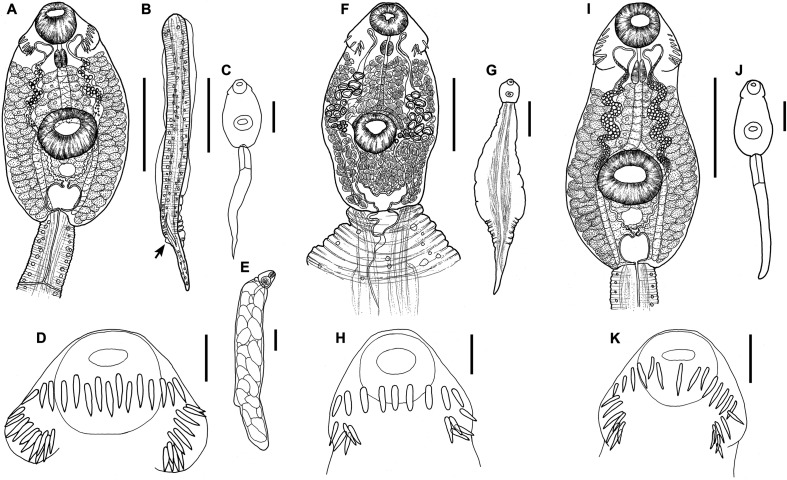
Drawings of cercariae. *Echinostoma revolutum* ex *Radix auricularia*. (A) body, ventral view, (B) tail with fin-folds, lateral view, (C) total view, (D) head collar with collar spines, (E) redia, lateral view. *Neopetasiger* sp. 5 ex *Planorbis planorbis*. (F) body, ventral view, (G) total view, (H) head collar with collar spines. Echinostomatidae gen. sp. ex *Lymnaea stagnalis*. (I) body, ventral view, (J) total view, (K) head collar with collar spines. Scale-bars: A, B, C, F, I, 100 μm; D, H, K, 20 μm; E, G, J, 200 μm.

**Figure 7:**
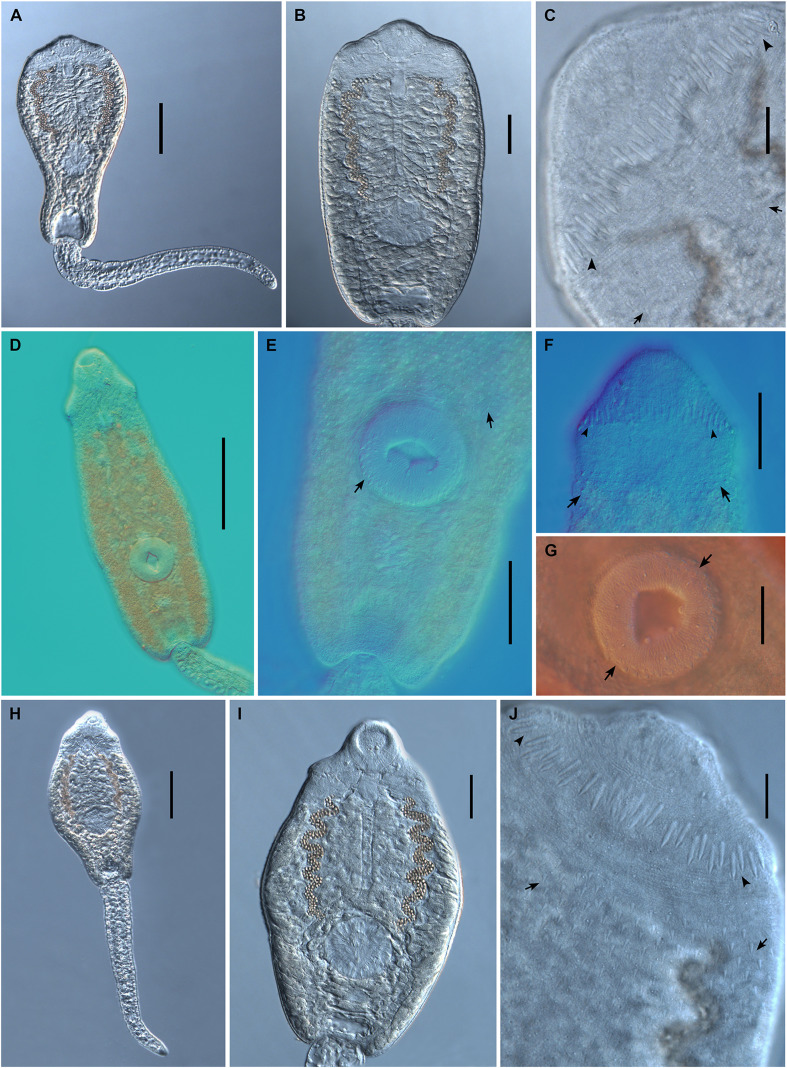
Photomicrographs of live cercariae of *Echinoparyphium recurvatum* ex *Radix balthica*. (A) body and tail, ventral view, (B) body, ventral view, (C) head collar, dorsal view, showing dorsal collar spines (arrowheads) and tegumental spines (arrows). Fixed cercariae of *Echinoparyphium* sp. 1 ex *Valvata macrostoma*. (D) total view, (E) ventral view with ventral sucker with tegumental spines and tegumental spines on body, (F) head collar, dorsal view, showing dorsal collar spines (arrowheads) and tegumental spines (arrows), (G) detail of ventral sucker with tegumental spines (arrows). Live cercariae of *Echinoparyphium* sp. 2 ex *Physa acuta*. (H) body and tail, ventral view, (I) body, ventral view, (J) head collar, dorsal view, showing dorsal collar spines (arrowheads) and tegumental spines (arrows). Scale bars: A, D, E, F, G, H, 100 μm, B, I, 50 μm, C, J, 20 μm.

(Description and measurements based on live material of six specimens; not all specimens contributed a data point to all metrical variables): Body elongate-oval, with maximum width just anterior to ventral sucker, 327–376 × 168–201 (351 × 185). Tegument thick, armed with robust, triangular, sharply pointed tegumental spines (see arrows Fig. 7C), 4–6 (5) long, becoming more slender and sharper posteriorly; extending ventrally from behind collar up to half-distance between ventral sucker and posterior body extremity; dorsally extending up to anterior level of ventral sucker. Collar well developed, 58–79 × 110–141 (68 × 126), with 45, slender, sharply pointed collar spines (see Fig. 5C, arrowheads Fig. 7C). Collar spine arrangement: on each side four angle spines, 12–16 (14) long; 37 marginal spines in double row; lateral spines 12–16 (14) long; dorsal aboral spines 12–16 (14), dorsal oral spines 10–15 (12) long, smaller than aboral spines (see Fig. 5C). Oral sucker ventro-subterminal, rounded, muscular, 41–56 × 48–64 (49 × 55). Ventral sucker rounded to transversely-oval, muscular, postequatorial, outer margin surrounded by small inconspicuous tegumental fold, 53–70 × 53–82 (61 × 68), larger than oral sucker; sucker width ratio 1:0.97–1.51 (1.23). Prepharynx distinct, narrow, highly contractile, slightly shorter than pharynx, 4–16 (10) long. Pharynx oval to elongate-oval, muscular, 24–33 × 16–28 (28 × 21). Oesophageal primordium long; intestinal bifurcation anterior to ventral sucker. Caecal primordia reach to anterior level of excretory vesicle or up to posterior body extremity. Cystogenous gland-cells numerous, with fine granular contents, extending from posterior level of pharynx to posterior extremity of body, most prominent in two lateral and one median field. Penetration gland-cells indistinct, around oesophageal primordium, stain slightly with Neutral red, number could not be determined, outlets on dorsal lip of oral sucker. Genital anlagen consist of two compact, interconnected, transparent groups of small cells, located median, anterodorsal and posterior to ventral sucker. Excretory vesicle saccular, rounded, constricted anteriorly. Main collecting ducts ascending from constricted part of excretory vesicle, ducts dilated between level of pharynx and anterior level of ventral sucker, densely filled with c. 105–200 small refractive excretory granules of similar size, diameter 3–5 (4), becoming smaller only anteriorly and posteriorly; ducts narrow and reflex at level of pharynx and lead backwards. Flame-cell formula 2[(3 + 3 + 3) + (3 + 3 + 3 + 3 + 3)] = 48. Excretory pore at junction of body and tail; caudal excretory duct bifurcates at c. the first quarter of tail length into two oblique branches opening laterally. Tail simple, devoid of fin-folds, of similar length as body or longer when live, muscular, contractile, with bluntly pointed tip, 399–489 × 45–67 (447 × 55).

Measurements of cercariae fixed in cold formalin (based on 25 specimens; not all specimens contributed a data point to all metrical variables): Body 219–319 × 123–196 (275 × 147). Collar 46–73 × 76–118 (59 × 90). Oral sucker 35–52 × 39–54 (44 × 47). Ventral sucker 41–67 × 48–82 (54 × 62). Sucker width ratio 1:1.02–1.58 (1:1.31). Prepharynx 3–14 (8) long. Pharynx 18–20 × 13–21 (23 × 16). Tail 269–451 × 36–53 (383 × 44). TL/BL ratio 0.96–1.73 (1.40).

Redia (Fig. 5D)

(Description and measurements based on 10 specimens of live daughter-rediae ex *R*. *balthica* from Finland): Body with orange-brownish pigment, elongate, tapered anteriorly and posteriorly, 2100–3575 × 300–375 (2935 × 330). Collar well pronounced, entire, slightly narrower than body. Birth pore just posterior to collar. Two prominent locomotory appendages present at about 2/3 of body length. Pharynx large, rounded, muscular, 300–400 × 325–425 (365 × 383). Intestine short, sac-like, in c. first fifth of body.

*Remarks:* The morphology of the present cercariae agrees well with that of the genus *Echinoparyphium* Dietz, 1909 in the presence of sharply pointed collar spines, four angle spines, marginal spines arranged in a double row, dorsal spines differing in size, tail devoid of fin-folds, numerous (>100) and relatively small excretory granules (<6 μm) in main collecting ducts [34, 53]. Following the key of Faltýnková et al. [34], the cercariae in our material key down to *E. recurvatum* in the presence of 45 collar spines and in the size of body (being close to the range of 200–250 μm) as well as the characters listed above. Grabda-Kazubska & Kiseliene [53] who redescribed the cercaria of *E. recurvatum* ex *Radix* spp. and distinguished it from the form occurring in planorbid snails (*E. pseudorecurvatum* Kiseliene & Grabda-Kazubska, 1990), consider the description of *E. recurvatum s. str.* ex *Lymnaea peregra* (sic) provided by Rašín [111] as the most comprehensive, and they view his material as the basis for recognition of *E. recurvatum s. str.*, because Rašín [111] completed the whole life-cycle of this species based on material from nature and from experiments.

The morphology of our cercariae agrees well with the description of Rašín [111], i.e. the dorsal oral collar spines are smaller than the dorsal aboral spines; the angle spines correspond in size (12–16 μm vs. 12–14 μm), while the aboral (12–16 μm vs. 13 μm) and oral spines (10–15 μm vs. 11 μm) are slightly larger. The excretory granules correspond in arrangement and size (3–5 μm vs. 5 μm). Also, the dimensions of cercariae (size of body, tail and collar) measured live agree. Our cercariae also agree with those described by Grabda-Kazubska & Kiseliene [53] in morphology of body and arrangement and size of collar spines and size of tegumental spines (4–6 μm vs. 5 μm). We update the distribution of tegumental spines, which are extending behind the ventral sucker ventrally and reach up to half-distance between ventral sucker and posterior extremity; dorsally they extend to the anterior level of the ventral sucker. There were different reports on the extent of the tegumental spines [111] as it is difficult to observe the minute spines in the posterior part of the body. Only the body length provided by Grabda-Kazubska & Kiseliene [53] is slightly larger (319–420 (367) μm) than our live and formalin fixed material, which could be due to the method of fixation (cercariae heat fixed and a drop of formalin added in [53]).

*Echinoparyphium recurvatum* and its life-cycle has been reported since the 1920’s (see [53] for citations) resulting in many records from a wide variety of hosts from all over the world. There arose doubts about its identity, and already Odening [103, 104] and Grabda-Kazubska & Kiseliene [53] claimed that the cosmopolitan *E. recurvatum* contained more than one species. Although Grabda-Kazubska & Kiseliene [53] delineated *E. recurvatum* and clarified the identity of the species, there has so far been no description linked to molecular identification (i.e. Soldánová et al. [122], Kostadinova et al. [76], Tkach et al. [126] provided no morphological descriptions). Therefore, for the first time, we provide a morphological description linked to DNA sequence data, and we corroborate its wide distribution in Europe.

Grabda-Kazubska & Kiseliene [53] who examined the chaetotaxy of *E*. *recurvatum*, found that the chaetotaxy of *E. recurvatum* ex *R. ovata* and *L. stagnalis* reported in Nezvalová [100] showed the same pattern; however, the groups of sensillae seemed to be incomplete. Therefore, they could not compare them fully with their material, and so we cannot conclude with certainty if *E. recurvatum* occurs in *L. stagnalis*. We only found *E. recurvatum* in *R*. *balthica* and *M*. *glutinosa*. Frolova [40] reported *E. recurvatum* from Karelia (Russia) in the snails *M. glutinosa*, *Stagnicola palustris* (O. F. Müller, 1774) and *Radix ovata* (syn. of *R. balthica*).

###### *Echinoparyphium rubrum* (Cort, 1917)

*First intermediate host*: *Stagnicola elodes* (Say) (Gastropoda: Lymnaeidae).

*Locality*: Tanana, pool on riverbank, Alaska, USA.

*Representative DNA sequences*: MZ404658, MZ404659 (*nad*1); MZ409805, MZ409806 (*28S*).

**Figure 8:**
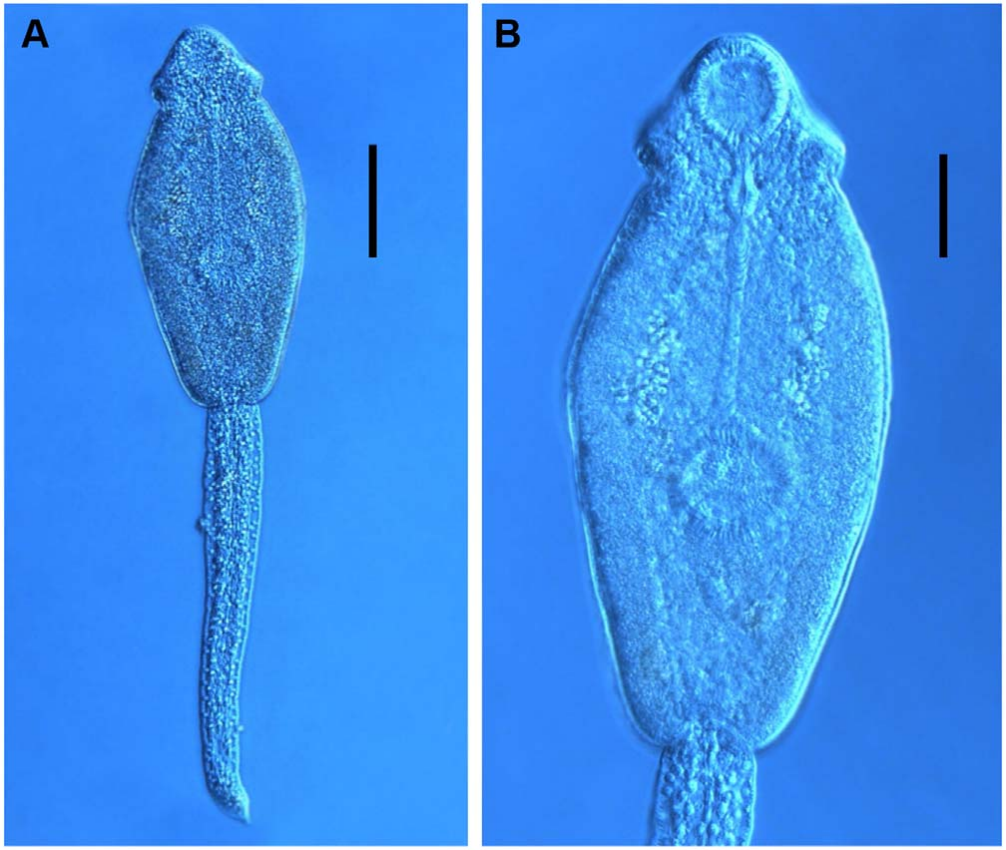
Photomicrographs of fixed cercariae of *Echinoparyphium rubrum* ex *Stagnicola elodes*. (A) total view, (B) body, ventral view. Scale bars: A, 100 μm, B, 50 μm.

Cercaria (Figs. 5E–5G, 8A–8B)

(Description based on live material and 16 formalin fixed specimens): Body elongate-oval, with maximum width at anterior level of ventral sucker. Tegument thick, armed with robust, triangular, tegumental spines, partly embedded in tegument, becoming smaller and sharper posteriorly; ventrally extending to half distance between posterior level of ventral sucker and posterior extremity, dorsally extending up to posterior level of ventral sucker. Collar well developed, with 43, relatively robust, sharply pointed collar spines with short tip. Collar spine arrangement: on each side four angle spines; 35 marginal spines in double row, dorsal spines slightly smaller than lateral spines. Oral sucker ventro-subterminal, rounded, muscular. Ventral sucker rounded to transversely-oval, muscular, postequatorial, with a circle of six papillae, larger than oral sucker. Prepharynx very short. Pharynx oval to elongate-oval, muscular. Oesophageal primordium long; intestinal bifurcation just anterior to ventral sucker. Caecal primordia reach to posterior body extremity. Cystogenous gland-cells numerous, with fine granular contents, extending from posterior level of pharynx to posterior extremity of body. Penetration gland-cells indistinct, around oesophageal primordium, number could not be determined. Genital anlagen consist of two compact, interconnected, transparent groups of densely arranged cells, located median, anterodorsal and posterior to ventral sucker. Excretory vesicle saccular, rounded, constricted anteriorly. Main collecting ducts ascending from smaller constricted part of excretory vesicle, ducts dilated between level of pharynx and anterior level of ventral sucker, densely filled with 80–86 small refractive excretory granules of similar size (for diameter see below), becoming smaller only anteriorly and posteriorly; ducts narrow and reflex at level of pharynx and lead backwards. Flame-cell formula not determined. Excretory pore at junction of body and tail; caudal excretory duct bifurcates at c. first quarter of tail length into two oblique branches opening laterally. Tail simple, devoid of fin-folds, longer than body when fixed, muscular, contractile, with short, pointed tip.

Measurements of cercariae fixed in cold formalin (based on 16 specimens; not all specimens contributed a data point to all metrical variables): Body 295–400 × 135–174 (337 × 155). Collar 64–113 × 81–120 (70 × 103). Oral sucker 49–60 × 46–59 (55 × 52). Ventral sucker 56–72 × 58–74 (64 × 64). Sucker width ratio 1:1.13–1.39 (1:1.25). Prepharynx 3–9 (6) long. Pharynx 18–31 × 15–26 (25 × 18). Collar spines: angle spine 15 long; lateral spines 10–14 (12) long; dorsal spines 11–12 (11) long. Diameter of excretory granules 3–5 (4). Tail 303–439 × 44–58 (391 × 50). TL/BL ratio 1.10–1.40 (1.26).

*Remarks*: The present cercariae agree well in their morphology with those of the genus *Echinoparyphium* in the presence of 43 collar spines with four angle spines and in the presence of numerous (>80), relatively small (<6 μm) excretory granules and a simple tail devoid of fin-folds, longer than body [73]. *Cercaria rubra* Cort, 1914 was first recorded by Cort [21] as metacercariae in the snail *Campeloma subsolidum* Antony (Viviparidae) in Connecticut, USA, therefore it is impossible to compare the original material. The entire life-cycle of *E*. *rubrum* has been elucidated experimentally and all developmental stages were described by Kanev et al. [67], alas, without molecular genetic analyses. The general morphology of the present cercariae is similar to those described by Kanev et al. [67], who obtained them experimentally from *Physa gyrina* Say and *P. occidentalis* (syn. of *P. gyrina*). The body dimensions of our cercariae (fixed in 4% formalin solution) are similar to those provided by Kanev et al. [67]; the size of body overlaps, although the body length in our material is slightly shorter (295–400 μm vs. 380–550 μm) as well as the tail length (303–439 μm vs. 390–560 μm); however, the prepharynx in our material is much shorter (3–9 μm vs. 30 μm).

Metacercariae of *E*. *rubrum* were recorded by Pulis et al. [109] from the wood frog, *Lithobates sylvaticus* (LeConte) in the Northern Great Plains, and cercariae from *Helisoma trivolvis* were recorded by Tkach et al. [129] in Minnesota, USA. Gordy & Hanington [51] recorded cercariae (*nad*1, matching our sequences of *E. rubrum*, see above) in lakes in Alberta, Canada, thus indicating that this species is most probably common and widely distributed in the northern USA and in Canada up to Alaska. As evidenced by previous and the present study, surprisingly, the first intermediate host spectrum is encompassing three families of snails, i.e. the physid *Physa* spp. obtained experimentally by Kanev et al. [67], the planorbid *Helisoma* [129] and the lymnaeid *S*. *elodes* in the present study.

###### *Echinoparyphium* sp. 1

*First intermediate host*: *Valvata macrostoma* Mörch (Gastropoda: Valvatidae).

*Locality*: Lake Konnevesi, Finland.

*Representative DNA sequences*: MZ404660–MZ404662 (*nad*1); MZ409807 (*28S*).

Cercaria (Fig. 5H–5J, 7D–7G)

(Description based on live material and six formalin fixed specimens): Body elongate-oval, maximum width just anterior to ventral sucker. Tegument thick, covered with short, robust, triangular, bluntly pointed tegumental spines becoming smaller posteriorly; spines extending from some distance posterior to collar to posterior level of ventral sucker ventrally; dorsally extending to anterior level of ventral sucker. Collar well developed, with 45, stout, relatively short spines with short and sharply pointed tip; all collar spines of similar size, dorsal spines in double row. Collar spine arrangement: on each side four angle spines and six lateral spines, dorsal spines 25. Oral sucker ventro-subterminal, rounded, muscular. Ventral sucker rounded, muscular, post-equatorial, slightly larger than oral sucker, outer surface covered by inconspicuous tegumental fold bearing 2–3 rows of sharp-pointed tegumental spines; with an outer circle of six smaller papillae, and with an inner circle of four larger papillae (Fig. 7G). Prepharynx nearly as long as pharynx. Pharynx muscular, oval. Oesophageal primordium long; intestinal bifurcation just anterior to ventral sucker. Caecal primordia consist of single rows of cells with granular content, reach to posterior extremity of body. Cystogenous gland-cells numerous, rounded, with fine granular contents, extending from posterior level of pharynx to posterior extremity of body, most prominent laterally. Penetration gland-cells arranged on both sides of oesophageal primordium, of five pairs, ducts opening on dorsal lip of oral sucker, slightly stain with Neutral red. Genital anlagen consist of two oval, compact, interconnected groups of small transparent cells, anterodorsal and just posterior to ventral sucker. Excretory vesicle saccular, rounded (can enlarge up to width of ventral sucker), constricted anteriorly. Main collecting ducts ascending from constricted part of excretory vesicle, dilated between posterior level of pharynx and anterior level of ventral sucker, densely filled with numerous (c. 160) refractive excretory granules of similar size (for diameter see below), in most specimens simple, in some specimens 2–3 granules may fuse; ducts narrow and reflex at level of pharynx and lead backwards. Flame-cells arranged in triplets, flame-cell formula 2[3 + 3+(3 + 3+3)] = 30; numerous ciliary tufts in main channels, c. eight on each side. Excretory pore at junction of body and tail; caudal excretory duct bifurcates in c. the first fifth of tail length. Tail muscular, simple, of similar length as body when live; with blunt tip.

Measurements of formalin fixed cercariae (based on six specimens; not all specimens contributed a data point to all metrical variables): Body 346–384 × 109–127 (365 × 119). Collar 60–78 × 80–102 (72 × 93). Oral sucker 43–47 × 43–63 (45 × 51). Ventral sucker 44–59 × 45–61 (52 × 53). Sucker width ratio 1:0.97–1.13 (1:1.05). Prepharynx 19–27 (21) long. Pharynx 18–22 × 19–25 (20 × 21). Collar spines: angle spines 9–11 (10) long, lateral spines 10–12 (11), dorsal spines 8–11 (9). Tegumental spines 2–3 (3) long. Diameter of excretory granules 3–4 (3). Tail 359 × 27. TL/BL ratio 0.96.

Rediae (Fig. 5K)

(Measurements of live daughter-rediae, based on 10 specimens): Body elongate, orange-yellowish, 828–1908 × 105–141 (1242 × 119). Collar well pronounced, entire. Birth pore just behind collar. Two prominent locomotory appendages present postequatorially (i.e. in third quarter of body length), 34–56 (41) long. Pharynx rounded, muscular, 53–70 × 46–63 (63 × 55). Intestine sac-like, with red-brownish contents, in c. first quarter of body.

*Remarks*: The present cercariae agree well in their morphology with *Echinoparyphium* in the characters as mentioned above [73]. The present species is genetically close to *E. mordwilkoi*, based on sequences provided by Stanevičiūtė et al. [123]; however, they were without morphological documentation. Also, in morphology, the present cercariae are similar to *E. mordwilkoi* which was characterised morphologically by Grabda-Kazubska & Kiseliene [54] ex *Valvata piscinalis* (O. F. Müller) in Lithuania (Lake Asveja). The dimensions of the cercariae overlap; however, the present cercariae differ in possessing clearly visible tegumental spines on the surface of the ventral sucker (Figs. 7E and 7G); also the ventral posterior extent of the tegumental spines is different (reaching the posterior level of the ventral sucker vs. to the half distance between the ventral sucker and posterior body extremity). Echinostome cercariae were reported from *V. piscinalis* in Great Britain by Harper [58] and McCarthy [93] as *Echinoparyphium recurvatum* which was revealed as a species complex [93], thus the identity of these records is unclear. Wesenberg-Lund [137] reported echinostome cercariae as *Cercaria abyssicola* Wesenberg-Lund, 1934 from *V. piscinalis* from Tjustrup Lake in Denmark; and Zdun [141] recorded *C. abyssicola* in *V. piscinalis* from the river Tisa in Ukraine. Also, Kiseliene et al. [72] recorded *C. abyssicola* ex *V. piscinalis* from Lake Asveja, Lithuania; however, they described the cercariae with a long fin-fold on tail, indicating that it belongs to a genus other than *Echinoparyphium*. The present cercariae are a species new to science and thus we confirm the diversity within the members of the genus *Echinoparyphium*, and the necessity to combine morphological and genetic data for accurate species delimitation.

###### *Echinoparyphium* sp. 2

*First intermediate host*: *Physa acuta* Draparnaud (Gastropoda: Physidae).

*Locality*: pond at Nordic House, Iceland.

*Representative DNA sequences*: MZ404663–MZ404665 (*nad*1); MZ409808 (*28S*).

Cercaria (Figs. 5L–5N, 7H–7J)

(Description and measurements based on live material of three specimens): Body elongate-oval, 312–347 × 203–232 (329 × 217), with maximum width just anterior to ventral sucker. Tegument thick, armed with robust, triangular, quite long, sharply pointed tegumental spines, those posterior to collar 3–5 (4) long; more slender and sharply pointed dorsally, becoming smaller and more slender posteriorly, particularly minute posterior to ventral sucker; ventrally extending from behind the collar up to posterior extremity, dorsally extending to forebody. Collar well developed, 60–79 × 108–117 (69 × 113), with 45, relatively slender, sharply pointed collar spines. Collar spine arrangement: on each side four angle spines 12–15 (14) long; 37 marginal spines in double row; lateral spines 12–13 (13) long; dorsal aboral spines 13–14 (14) long; dorsal oral spines 12–14 (13) long, of similar length as aboral spines but more slender. Oral sucker ventro-subterminal, rounded, muscular, 45–52 × 59–63 (48 × 61). Ventral sucker rounded to transversely-oval, muscular, postequatorial, surrounded by inconspicuous radial tegumental fold, with a circle of six papillae, 59–69 × 78–85 (63 × 81); larger than oral sucker; sucker width ratio 1:1.25–1.44 (1:1.33). Prepharynx distinct, narrow, 13–20 (17) long, up to length of pharynx. Pharynx oval to elongate-oval, muscular, 23–24 × 19–24. Oesophageal primordium long; intestinal bifurcation anterior to ventral sucker. Caecal primordia reach to posterior body extremity. Cystogenous gland-cells numerous, with fine granular contents, extending from posterior level of pharynx to posterior extremity of body. Penetration gland-cells indistinct, around oesophageal primordium, number could not be determined, inconspicuous outlets on dorsal lip of oral sucker. Genital anlagen consist of two compact, interconnected, transparent groups of small cells, located median, anterodorsal and posterior to ventral sucker. Excretory vesicle saccular, rounded, constricted anteriorly. Main collecting ducts ascending from constricted part of excretory vesicle, ducts dilated between level of pharynx and anterior level of ventral sucker, densely filled with c. 100–216 small refractive excretory granules of similar size, in most specimens simple, in some specimens 2–3 granules may fuse, diameter 3–5 (4), becoming smaller only anteriorly and posteriorly; ducts narrow and reflex at level of pharynx and lead backwards. Flame-cell formula not determined. Excretory pore at junction of body and tail; caudal excretory duct bifurcates at c. first quarter of tail length into two oblique branches opening laterally. Tail simple, devoid of fin-folds, longer than body when live, muscular, contractile, with bluntly pointed tip.

Measurements of cercariae fixed in ethanol (based on 30 specimens; not all specimens contributed a data point to all metrical variables): Body 304–396 × 127–180 (340 × 151). Collar 62–92 × 82–110 (75 × 96). Oral sucker 37–58 × 39–54 (48 × 47). Ventral sucker 40–71 × 48–74 (57 × 64). Sucker width ratio 1:1.04–1.69 (1:1.36). Prepharynx 8–22 (15) long. Pharynx 20–35 × 15–25 (26 × 19). Collar spines: angle spines 12–14 (13) long; lateral spines 11–13 (12); dorsal oral spines 11–12 (11); dorsal aboral spines 11–13 (12) long. Diameter of excretory granules usually 3–5 (4), in some specimens with fused granules, diameter up to 9. Tail 337–481 × 40–57 (440 × 48). TL/BL ratio 0.93–1.57 (1.31).

Redia (Fig. 5O)

(Measurements of live daughter-rediae, based on two specimens): Body stout, orange-brownish, with tapered anterior extremity, blunt posterior extremity, 1293–1727 × 371–373. Collar well developed, narrower than body, 163–233 wide. Birth pore just behind collar. Two locomotory appendages present at mid-level of body, 62 long. Pharynx small, rounded, muscular, 49–52 × 49–50. Intestine short, in c. first fifth of body, sac-like.

*Remarks*: The present cercariae agree in their morphology with those of the genus *Echinoparyphium* in characters stated above [62, 73], only in some specimens larger excretory granules were noticed (with a diameter up to 9 μm). Our new sequences for *nad*1 are highly similar to sequences deposited in GenBank by Gordy and Hanington [51] and identified as *Echinoparyphium* sp. A (MH369158 and MH369047), and *Hypoderaeum* sp. Lineage 2 (MH369080), all from *Physa gyrina* in Alberta, Canada. Alas, the same sequences were identified under different names by Gordy & Hanington [51], therefore, to avoid further confusion, we chose to name our species *Echinoparyphium* sp. 2. The present cercariae were found in one single locality in Iceland (Nordic House, Vatnsmýri bird reserve, Reykjavík), a popular area for nesting birds and with an established population of *P. acuta*, an invasive snail originating from North America [91], dwelling in Iceland for more than 40 years (Skírnisson & Schleich, unpublished). We had been finding *Echinoparyphium* sp. 2 consistently for the last two years in snails, indicating that the trematode’s circulation in the environment is well established. The relation to the North American trematodes recorded by Gordy & Hanington [51] underlines the link of the Icelandic fauna to that of North America presupposed by the occurrence of suitable intermediate hosts (introduced *P. acuta*) and migratory bird hosts nesting in north Canada and stopping or wintering in Iceland [125].

#### *Echinostoma* Rudolphi, 1809

##### *Echinostoma nasincovae* Faltýnková, Georgieva, Soldánová & Kostadinova, 2015

*First intermediate host*: *Planorbarius corneus* (Linnaeus) (Gastropoda: Planorbidae).

*Locality*: Lough Corrib, Ireland.

*Representative DNA sequences*: MZ404666 (*nad*1); MZ409809 (*28S*).

*Remarks*: Cercariae of *Ec*. *nasincovae* are among the most common in Europe [32, 35] in *P. corneus*; for a long time, they had been reported under the name *Cercaria spinifera* La Valette, 1855 or *Echinostoma spiniferum* [98, 99]. The natural definitive host is still unknown, and the life-cycle had been elucidated using birds (*Gallus gallus f. dom.*, *Anas platyrhynchos f. dom.*) and mammals (*Mesocricetus auratus*) as experimental hosts by Našincová [99] (see [32]). The species is a new record for Ireland, indicating its wide distribution also in the western part of Europe.

##### *Echinostoma revolutum* (Frölich, 1802) Rudolphi, 1809 *sensu stricto*

*First intermediate host*: *Radix balthica* (Linnaeus) (Gastropoda: Lymnaeidae).

*Locality*: pond at Nordic House, Iceland.

*Representative DNA sequences*: MZ404667–MZ404672 (*nad*1); MZ409810 (*28S*).

*Remarks*: Cercariae of *Echinostoma revolutum s. str.* have been occurring regularly in *R. balthica* in Iceland [46, 48], see Table 1); the species is common in Central Europe in different first intermediate hosts (*R. auricularia*, *R. balthica*, *L. stagnalis*, and *S. palustris*; [31, 46, 48]). Georgieva et al. [46] found *Ec. revolutum* in tufted duck *Aythia fuligula*; these and other ducks are also common in Iceland, thus ensuring the circulation of the life-cycle stages there. The species is the type-species of the so called “*revolutum*” group, the systematics of which had long been controversial, and which was resolved by Georgieva et al. [46] and Faltýnková et al. [32] in Europe as the species *sensu stricto*, and the species from North America was revealed as a closely related species, which still awaits description as a new species (see below).

##### *Echinostoma revolutum* (Frölich, 1802) Rudolphi, 1809 of Detwiler et al. [24]

*First intermediate hosts*: *Radix auricularia* (Linnaeus), *Stagnicola elodes* (Say) (Gastropoda: Lymnaeidae).

*Localities*: Fairbanks, small lake near airport, Tanana, pool on river bank, Alaska, USA.

*Representative DNA sequences*: MZ404673–MZ404675 (*nad*1); MZ409811 (*28S*).

**Figure 9:**
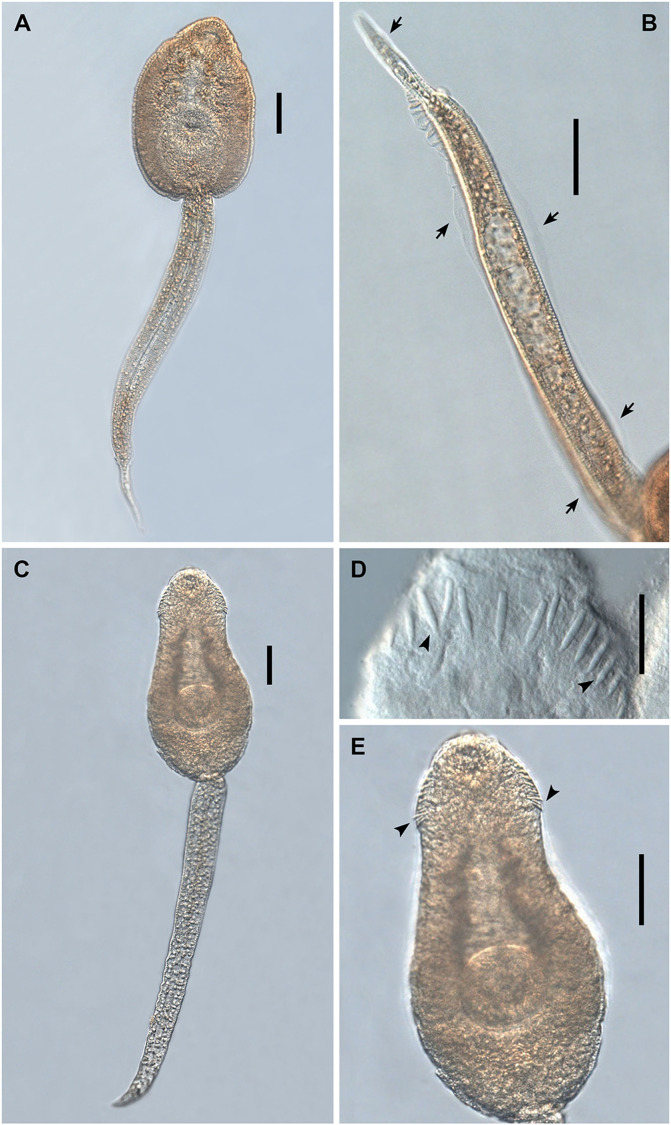
Photomicrographs of live cercariae of *Echinostoma revolutum* ex *Radix auricularia*. (A) body and tail, ventral view, (B) tail showing fin-folds (arrows), lateral view. Fixed cercariae of Echinostomatidae gen. sp. ex *Lymnaea stagnalis.* (C) body and tail, ventral view, (D) head collar, dorsal view, showing dorsal collar spines (arrowheads), (E) body, showing angle collar spines (arrowheads), ventral view. Scale-bars: A, B, E, 50 μm; C, 100 μm; D, 20 μm.

Cercaria (Figs. 6A–6D, 9A–9B)

(Description based on live material and 11 fixed specimens): Body oval to elongate-oval, muscular, with maximum width just anterior to ventral sucker. Tegument thick, armed with triangular spines, becoming smaller and less dense posteriorly; ventrally reaching up to posterior level of ventral sucker, dorsally reaching to anterior level of ventral sucker. Collar well developed, with 37, long, robust collar spines with sharply pointed tips, all of similar size (Fig. 6D). Collar spine arrangement: on each side five angle spines and six lateral spines in single row; 15 dorsal spines in double row. Oral sucker subterminal, muscular, rounded. Ventral sucker rounded, muscular, just postequatorial, larger than oral sucker. Prepharynx distinct, shorter than pharynx. Pharynx elongate-oval, muscular. Oesophageal primordium long; intestinal bifurcation at level of first quarter of ventral sucker. Caecal primordia reach to posterior body extremity. Cystogenous gland-cells numerous, with fine granular contents, occupy most of body posterior to pharynx. Penetration gland-cells indistinct, around oesophageal primordium, covered by cystogenous gland-cells; their duct openings present on dorsal lip of oral sucker. Paraoesophageal gland-cells few (probably 4–5 pairs, exact number could not be determined), located on both sides of oesophagus, with long ducts, outlets surrounding oral sucker. Genital anlagen consist of two compact, interconnected, transparent groups of small cells, located median, anterodorsal and posterior to ventral sucker. Excretory vesicle saccular, constricted anteriorly. Main collecting ducts ascending from constricted part of excretory vesicle, dilated between posterior level of pharynx and anterior margin of ventral sucker, contain c. 30–40 simple or double refractive excretory granules of different size, largest at mid-level of ducts (for diameter see below); ducts narrow and reflex at level of prepharynx and lead backwards. Flame-cell formula not determined. Excretory pore at junction of body and tail. Caudal excretory duct bifurcates in c. first quarter to first fifth of tail length into two oblique branches opening laterally. Tail muscular, contractile, longer than body when fixed; tip forms highly contractile slender process (nearly 1/5 of tail length). Seven well-pronounced tegumental fin-folds present on tail: two dorsal, three ventral and two ventro-lateral (Figs. 6B and 9B). Distal dorsal and ventral fin-folds most prominent, distal dorsal fin-fold longer than ventral one; distal ventral fin-fold connected with proximal ventral fin-fold by narrow tegumental elevation; proximal dorsal fin-fold longest; ventro-lateral fin-folds short; smallest (but prominent) ventral fin-fold located near base of tip of tail.

Measurements of cercariae fixed in cold formalin (based on 11 specimens; not all specimens contributed a data point to all metrical variables): Body 185–239 × 123–150 (210 × 136). Oral sucker 42–51 × 41–48 (46 × 46). Ventral sucker 44–64 × 56–69 (56 × 64). Sucker width ratio 1:1.21–1.50 (1:1.40). Prepharynx 2–5 (4) long. Pharynx 13–23 × 11–18 (18 × 15). Collar spines: angle spines 9–14 (12) long; lateral spines 9–14 (12); dorsal spines 10–14 (12). Tegumental spines 2–3 (2) long. Diameter of excretory granules 3–7 (5). Tail 345–439 × 36–48 (405 × 43). TL/BL = 1.65–2.17 (1.93).

Redia (Fig. 6E)

(Description and measurements of daughter-rediae fixed in cold formalin, based on 11 specimens, not all specimens contributed a data point to all metrical variables): Body brownish-orange, long, stout, 1674–3001 × 266–381 (2202 × 302). Collar well pronounced, entire, 125–152 (138) wide, narrower than body. Birth pore just posterior to collar. Two prominent locomotory appendages present in second half of body, 65–79 (71) long. Pharynx rounded, muscular, 55–61 × 49–55 (58 × 52). Intestine short, sac-like, with reddish contents, reaching not far behind collar.

*Remarks*: The morphology of the present cercariae agrees well with those of the genus *Echinostoma* in the presence of 37 collar spines (five angle spines, dorsal spines in double row), a tail bearing seven fin-folds, and not too numerous (less than 50) excretory granules of differing size (largest ones up to 7 μm) [32, 34, 73]. The specimens described here belong to the lineage of *Ec. revolutum* as defined by Georgieva et al. [48]. The isolates recorded previously from North America by Detwiler et al. [24, 25] were shown by Georgieva et al. [48] to represent another (cryptic) species of the “*revolutum*” complex, i.e. *Ec. revolutum* and forming a sister clade to *Ec. revolutum s. str.* [48]. Morphologically, the present cercariae look similar to *Echinostoma revolutum s. str.* ex *L. stagnalis* from Europe in the presence of paraoesophageal gland cells with long ducts and also in the dimensions of the cercariae, which are similar. However, the cercariae differ in the arrangement of fin-folds on tail, which is similar to *Ec. paraulum* (another species belonging to the “*revolutum*” complex; [32]) in the connection of the ventral proximal and distal fin-folds by a small tegumental ridge. In North America, Beaver [5] described in detail cercariae of *Echinostoma revolutum*; however, they possess a larger and more slender body (323 × 95 μm), when fixed, and a slightly longer tail (450 μm) than cercariae in our samples. Also, the body spination is different, Beaver [5] stated that the whole body is spined ventrally and almost all dorsally (vs. spines reaching the posterior level of the ventral sucker ventrally and the anterior level of the ventral sucker dorsally in our material).

The present cercariae constitute a separate lineage, differing genetically and morphologically from *Ec. revolutum s. str.*; however, a description of the species awaits the discovery of adults. They use more than one lymnaeid snail species as first intermediate hosts, i.e. *S. elodes* widely distributed in USA and Canada [15], and *R. auricularia* which was introduced from Europe to North America [15, 133]. Moreover, because of the cryptic nature of the species of *Echinostoma*, further investigations on the diversity of *Echinostoma* based on integrative taxonomy will help to better evaluate the host-use and geographical distribution of *Echinostoma* in America.

##### *Echinostoma* sp. IG *sensu* Georgieva et al. (2013)

*First intermediate hosts*: *Radix balthica* (Linnaeus), *Myxas glutinosa* (O.F. Müller) (Gastropoda: Lymnaeidae).

*Localities*: Lake Ashildarholtsvatn, pond at Nordic House, Iceland; Lough Corrib, Ireland.

*Representative DNA sequences*: MZ404676–MZ404678 (*nad*1); MZ409812, MZ409812 (*28S*).

*Remarks*: The morphology of our new material from Iceland and Ireland corresponds well to *Echinostoma* sp. IG described by Georgieva et al. [48]. *Echinostoma* sp. IG was previously found in Iceland (in *R. balthica*), Germany (*R. auricularia*) and Great Britain (in *Planorbis* sp.) [48]; it is a new record for Ireland, indicating that this species is common in the European North Atlantic region as it is most probably circulating with aquatic birds nesting in Iceland and wintering in Ireland, Great Britain and continental Europe, moreover, the snail species used as first intermediate hosts are commonly distributed in Europe, while *R*. *balthica* is also distributed in Siberia and Central Asia [133].

#### *Hypoderaeum* Dietz, 1909

##### *Hypoderaeum conoideum* (Bloch, 1782) Dietz, 1909

*First intermediate host*: *Lymnaea stagnalis* (Linnaeus) (Gastropoda: Lymnaeidae).

*Locality*: Huumonjärvi, Finland.

*Representative DNA sequences*: MZ404679–MZ404682 (*nad*1); MZ409814 (*28S*).

*Remarks*: The new material from Finland keys down to *Hypoderaeum conoideum* of Faltýnková et al. [34]. The *nad*1 sequences of this species showed low intraspecific divergence to the sequences of *H*. *conoideum* published by Kostadinova et al. [76] and Miquel et al. [95]. The *28S* sequences of the present study were identical to those obtained from adults of *H. conoideum* in the USA and Ukraine published by Tkach et al. [126]. In the past, *H. conoideum* was reported also from Iceland by Blair [7] who found metacercariae in *Radix peregra* (syn. of *R. balthica*) and obtained adults experimentally which he identified as *H. conoideum*. In Europe, *H. conoideum* is a frequent parasite of anseriform birds, and as first intermediate hosts, common snail species were recorded, i.e. *L*. *stagnalis*, *Radix peregra* and *R. peregra ovata* (synonyms of *R. balthica*) and *S*. *palustris* [119].

#### *Moliniella* Hübner, 1939

##### *Moliniella anceps* (Molin, 1859) Hübner, 1939

*First intermediate host*: *Stagnicola fuscus* (C. Pfeiffer) (Gastropoda: Lymnaeidae).

*Locality*: Lough Mask, Ireland.

*Representative DNA sequences*: MZ404683 (*nad*1); MZ409815 (*28S*).

*Remarks*: The new material from Ireland corresponds well in morphology to *M*. *anceps* as in the key of Faltýnková et al. [34]. The only sequence (*28S*) of *M. anceps* available in GenBank was provided by Tkach et al. [126] of a metacercaria ex *P. corneus* in Lithuania. This one and our *28S* sequences were identical. Cercariae of this species were found not to be too common in *Stagnicola corvus* and *L. stagnalis* in central Europe; however, metacercariae are quite common [34]. The present study provides the first *nad*1 sequence for *M*. *anceps* and the first record of this species in Ireland, which is the westernmost distribution of the species.

#### *Neopetasiger* Bashkirova, 1941

##### *Neopetasiger islandicus* Kostadinova & Skírnisson, 2007

*First intermediate host*: *Gyraulus* cf. *parvus* (Say) (Gastropoda: Planorbidae).

*Localities*: lakes Ashildarholtsvatn and Mývatn, Iceland.

*Representative DNA sequences*: MZ404684–MZ404686 (*nad*1); MZ409816 (*28S*).

*Remarks*: The morphology of the present species corresponds well to that described by Georgieva et al. [47]. The adults of *Neopetasiger islandicus* were first described by Kostadinova & Skírnisson [77] from the horned grebe *Podiceps auritus* in Mývatn, Iceland. The other life-cycle stages were described by Georgieva et al. [47] ex *Gyraulus* cf. *laevis* and the three-spined stickleback, *Gasterosteus aculeatus*, respectively, from Lake Mývatn in Iceland. Since we have material of *N*. *islandicus* from the type-locality, we assume the correct identification of the snail host is *G*. cf. *parvus*, which is a species widely distributed in North America, and which was also found in south Greenland; currently it is spreading in Europe [50, 90, 135]. Adults and cercariae of *N*. *islandicus* were also found in North America in the western grebe *Aechmophorus occidentalis* (Lawrence, 1858), and the snail *Planorbula armigera* (Say, 1821), respectively [52, 126], indicating that the species is not restricted to Iceland, and it can be expected to occur throughout North America, as *A. occidentalis* occurs from British Columbia to California [30], and the snail *G*. *parvus* is common [15].

##### *Neopetasiger* sp. 5

*First intermediate host*: *Planorbis planorbis* (Linnaeus) (Gastropoda: Planorbidae).

*Locality*: Lough Corrib, Ireland.

*Representative DNA sequences*: MZ404687 (*nad*1); MZ409817 (*28S*).

**Figure 10:**
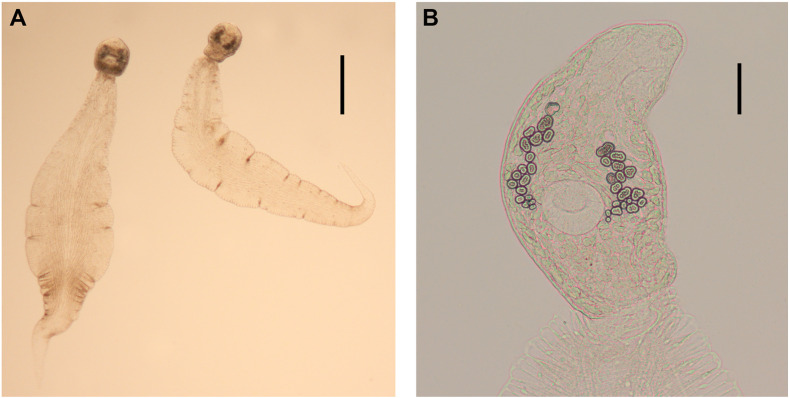
Photomicrographs of live cercariae of *Neopetasiger* sp. 5 ex *Planorbis planorbis*. (A) body and tail, ventral view, (B) body, ventral view. Scale-bars: A, 200 μm; B, 50 μm.

Cercariae (Figs. 6F–6H, 10A–10B)

(Description and measurements based on two live specimens): Body small, elongate-oval, dark, 195–203 long, with maximum width at level of ventral sucker, 114–121. Tegument thick, armed with minute spines embedded in tegument. Collar narrower than body, 47–67 × 80–97, well developed, with 19, robust, bluntly pointed collar spines. Collar spine arrangement: on each side four angle spines (14 long); two lateral spines on each side; seven dorsal spines (13–17 (14) long) in single row (see Fig. 6H). Oral sucker ventro-subterminal, rounded, muscular, 41–47 × 41–47. Ventral sucker rounded to transversely-oval, muscular, postequatorial, slightly larger than oral sucker, 51–52 × 52–61; sucker width ratio 1:1.12–1.47. Prepharynx distinct, narrow, of similar length as pharynx, 20–21 long; pharynx elongate-oval to oval, 18–24 × 18–22. Oesophageal primordium long; intestinal bifurcation anterior to ventral sucker. Caecal primordia narrow, reach to excretory vesicle. Cystogenous gland-cells numerous, with rhabditiform contents; extending from posterior level of pharynx to posterior extremity of body, most prominent in two lateral and one median row. Penetration gland-cells indistinct, around oesophageal primordium, number could not be determined. Genital anlagen consist of two interconnected transparent groups of small cells, anterodorsal and posterior to ventral sucker. Excretory vesicle bipartite, rounded at base; continues in anterior narrowed tail region as accessory excretory vesicle c. 22–29 long. Main ascending collecting ducts dilated between posterior level of pharynx and mid-level of ventral sucker, contain 18–29 large refractive excretory granules, formed by fusion of 2–4 smaller ones, granules of irregular shape, becoming smaller posteriorly, diameter 5–18; ducts narrow and reflex at level of prepharynx and lead backwards. Tail leaf-like (TL/TW ratio = 3.57–4.82), with strong musculature, almost transparent, with only few pigment, 1112–1122 long, much longer than body (TL/BL ratio 5.48–5.75), with maximum width at c. its mid-length, 231–314 wide.

Measurements of cercariae fixed in ethanol (based on 10 specimens): Body 223–243 × 104–118 (236 × 110). Collar 43–58 × 46–78 (52 × 67). Oral sucker 37–43 × 39–45 (40 × 43). Ventral sucker 44–62 × 47–56 (50 × 51). Sucker width ratio 1:1.09–1.35 (1:1.20). Collar spines: angle spines 13–15 (14) long; lateral spines 13–14 long. Diameter of excretory granules 4–16 (11). Tail 311–717 (536) long, strongly contracted when fixed, maximum width 97–240 (185), TL/TW ratio 2–4 (3); longer than body, TL/BL ratio 1.35–3.03.

*Remarks*: The morphology of the present cercariae corresponds well to that of the genus *Neopetasiger* in possessing a large, conspicuous tail, much longer than body, a total of 19 collar spines with four angle spines on each side, cystogenous gland-cells with rhabditiform contents and large composite excretory granules in main ascending excretory channels [118, 126]. In the keys of Kostadinova & Chipev [74] and Selbach et al. [118], the present cercaria keys down to *Cercaria tidfordensis* Nasir, 1962 ex *Planorbis carinatus* O.F. Müller in the presence of 19 collar spines, of a colourless body and tail, and a leaf-shaped tail, which is relatively short (311–717 μm vs. 540–740 μm; TL/TW ratio 2–4 vs. 3–4). However, the TL/BL ratio for fixed cercariae in our material is lower than in Kostadinova & Chipev [74] (TL/BL ratio 1.35–3.03 vs. 3.5–4.0). With the leaf-shaped tail, its most typical feature, our cercariae are similar to *N. islandicus*; however, the tail is shorter compared to *N. islandicus* (311–717 μm vs. 740–970 μm) and wider (TL/TW 2–4 vs. 4–8) and our cercariae possess less pigment, i.e. there is no yellow pigment in the body (vs. *N. islandicus*). Another leaf-shaped cercaria, *Cercaria thamesensis* of Khan [71] was described with 20 collar spines, and its tail is longer (TL/TW 4–9) than in our cercariae; therefore, it cannot be assigned to our material. Apparently, the diversity of *Neopetasiger* with cercariae with leaf-like tails is greater than is so far known and enlarges the number of yet provisionally-named species from snails to five (see Selbach et al. [118] for *Neopetasiger* sp. 1–4); however, a reliable description of a new species awaits the discovery of corresponding adults which most likely parasitise grebes.

#### Echinostomatidae gen. sp.

*First intermediate host*: *Lymnaea stagnalis* (Linnaeus) (Gastropoda: Lymnaeidae).

*Locality*: Huumonjärvi, Finland.

*Representative DNA sequences*: MZ404688, MZ404689 (*nad*1); MZ409818, MZ409819 (*28S*).

Cercaria (Figs. 6I–6K, 9C–9E)

(Description and measurements based on live material and 24 fixed cercariae): Body elongate-oval, with maximum width at level of ventral sucker. Tegument thick, armed with stout, triangular tegumental spines, not too dense, becoming smaller posteriorly; ventrally extending to mid-level of ventral sucker, dorsally extending to forebody. Collar well developed, with 27, long, slender sharply pointed collar spines. Collar spine arrangement: on each side four angle spines; four lateral spines in single row on each side; 11 dorsal spines with three aboral spines in centre and two smaller oral spines on each side, other dorsal spines aboral, in one row (see Figs. 6K and 9D). Oral sucker ventro-subterminal, rounded, muscular. Ventral sucker rounded, muscular, postequatorial, larger than oral sucker. Rim of both suckers surrounded by inconspicuous tegumental fold. Prepharynx distinct, narrow, nearly as long as pharynx. Pharynx oval, muscular. Oesophageal primordium long; intestinal bifurcation at level of anterior edge of ventral sucker. Caecal primordia reach to posterior body extremity. Cystogenous gland-cells numerous, with fine granular contents, extending from posterior level of pharynx to posterior extremity of body. Penetration gland-cells indistinct, around oesophageal primordium, number could not be determined. Genital anlagen consist of two compact, interconnected, transparent groups of small cells, located median, anterodorsal and posterior to ventral sucker. Excretory vesicle saccular, rounded, constricted anteriorly. Main collecting ducts ascending from constricted part of excretory vesicle, ducts dilated between level of pharynx and anterior level of ventral sucker, densely filled with 140–170 refractive excretory granules being largest in mid-part and becoming smaller anteriorly and posteriorly (for diameter see below); ducts narrow and reflex at level of pharynx and lead backwards. Flame-cell formula not determined. Excretory pore at junction of body and tail; caudal excretory duct bifurcates in c. first quarter of tail length into two oblique branches opening laterally. Tail simple, devoid of fin-folds, much longer than body when fixed, muscular, contractile, with bluntly pointed tip.

Measurements of formalin fixed cercariae (based on 10 specimens; not all specimens contributed a data point to all metrical variables): Body 267–379 × 125–157 (326 × 142). Collar 63–80 × 76–92 (69 × 85). Oral sucker 45–53 × 41–47 (50 × 44). Ventral sucker 49–61 × 55–59 (54 × 57). Sucker width ratio 1:1.21–1.41 (1:1.29). Prepharynx 6–19 (12) long. Pharynx 19–25 × 13–21 (22 × 16). Collar spines: angle spine 14 long; lateral spines 12–13 long; larger dorsal aboral spines 11–14 long; small dorsal oral spines 9–10. Tail 337–490 × 34–51 (440 × 44). TL/BL ratio 0.98–1.62 (1.37).

Measurements of ethanol fixed cercariae (based on 14 specimens; not all specimens contributed a data point to all metrical variables): Body 238–272 × 100–136 (254 × 118). Collar 51–86 × 57–83 (60 × 68). Oral sucker 35–48 × 36–48 (42 × 42). Ventral sucker 40–58 × 42–64 (48 × 52). Sucker width ratio 1:0.98–1.50 (1:1.26). Prepharynx 9–16 (13) long. Pharynx 15–22 × 11–18 (19 × 14). Diameter of excretory granules 3–6 (4). Collar spines: angle spines 8–14 (11) long; lateral spines 8– 14 (12) long; larger dorsal aboral spines 10–16 (13) long; smaller dorsal oral spines 8–12 (10) long. Tegumental spines posterior to collar 1–2 long. Diameter of excretory granules 3–6 (4). Tail 361–458 × 34–45(409 × 39). TL/BL ratio 1.48–1.88 (1.61).

*Remarks*: The present cercariae fall within the family Echinostomatidae in the presence of typical features stated above and in Kostadinova [73]. However, based on the combination of its characters, i.e. 27 slender collar spines, excretory granules of mid-size (up to 6 μm, similar to *Echinostoma*) and quite numerous (140–170 μm, typical for *Echinoparyphium*), we could not assign the present cercariae to any of the known genera of the family Echinostomatidae. The other genera with similar species bearing 27 collar spines are *Isthmiophora*, *Petasiger* (the former *Paryphostomum*), and *Drepanocephalus*; however, their cercariae have differently arranged collar spines (no smaller dorsal oral spines), suckers provided with a well-pronounced circular fin-fold and much larger and less numerous excretory granules. For the other known genera with species with 27 collar spines [73], such as *Bashkirovitrema*, *Chaunocephalus* and *Balfouria*, their cercariae are not documented, neither are they genetically sequenced. Although sequences of adult *Chaunocephalus* are available (Table 4), our isolate clustered in a distant clade from a clade with *C. ferox* (Rudolphi, 1795) (Fig. 4). Apart from the specific arrangement of the collar spines, which most resembles *Isthmiophora*, the excretory granules in our cercariae are similar to those of the genus *Echinostoma*, i.e. they are of a similar size and they are larger at the mid-level of the channels; however, they are more numerous and they are filling the channels as densely as in *Echinoparyphium*.

Although the morphology of the present cercariae is quite characteristic (smaller dorsal oral spines and excretory ducts densely filled with excretory granules larger than in *Echinoparyphium*), it is difficult to find a description precise enough for comparison. Our cercariae are most similar to those described by Ginetsinskaya & Dobrovolskiy [49] as *Cercaria helvetica* XXI Dubois, 1929 ex *Radix auricularia*, *R. ovata*, and *R. peregra* from Astrakhan, Russia, with 27 collar spines (however, with no precise information on collar spine arrangement) and main ducts filled with numerous small granules, with bigger ones in the middle. The dimensions of body (267–379 × 125–157 μm vs. 270–330 × 150–190 μm) in our material fixed in formalin are similar; however, the tail length (361–458 μm vs. 480–570 μm) and suckers in our cercariae are smaller (oral sucker 35–48 × 36–48 μm vs. diameter 50–70 μm; ventral sucker 40–58 × 42–64 μm vs. diameter 60–80 μm). Another similar cercaria described by Ginetsinskaya & Dobrovolskiy [49] ex *L. stagnalis* is *Cercaria astrakhanica VI* with 27 collar spines and larger excretory granules; however, these cercariae were much larger (body 410–550 μm) than those in our material. Since no further information on the collar spines is available and the drawings of excretory granules are quite schematic, it is impossible to say if the present cercariae could be identical with those described. Ginetsinskaya & Dobrovolskiy [49] recorded two more cercariae (*Cercaria astrakhanica* V and *Cercaria coronata* Kotova, 1939) with 27 collar spines, however with a differing size and number of excretory granules and with different metrical characteristics.

Another unidentified echinostome cercaria was described by Odening [104] as bearing usually 29 (sometimes 27–31) collar spines, from *L. stagnalis* in Germany and as occurring very rarely. The body length of these cercariae (257–294 μm) overlaps with that of our material, the tail (length 580–694 μm) however, is longer. Cercariae with the same morphology as in the present material were found in the Czech Republic before by one of the authors (AF, unpublished), indicating that these unidentified cercariae are likely a part of the European echinostome fauna; however, they are probably very rare. Only with further availability of molecular data involving more genera, can the systematic affiliation of this material be solved.

## Discussion

Using an integrative taxonomic approach and existing DNA sequence libraries, our investigation of the diversity of echinostomes in snails at more northern latitudes reports the presence of 14 species. Despite the fact that the diversity of echinostomes is relatively well studied in Europe [32, 46], we discovered four species, namely, *Echinoparyphium* sp. 1, *Echinoparyphium* sp. 2, *Neopetasiger* sp. 5 and Echinostomatidae gen. sp. that appear to be novel in Europe and unique to Finland (*Echinoparyphium* sp. 1, Echinostomatidae gen. sp.), Iceland (*Echinoparyphium* sp. 2) and Ireland (*Neopetasiger* sp. 5). Our survey on echinostomes revealed records of two species in Alaska (USA), five species each in Finland and Iceland, and six species in Ireland. All species found in Ireland are new records for this country. The present study shows that there is some connection of the American trematode fauna with that from Europe (Eurasia) in Iceland, and the life-cycles of the trematodes are well established on the island, which is enabled by the presence of the snail intermediate hosts and the migration of birds within the East Atlantic flyway.

Our study adds new data on the geographical distribution for several species, some of them well-known. For four European species, *E*. *aconiatum*, *E*. *recurvatum*, *Ec*. *nasincovae*, and *M*. *anceps* described and reported in central, southern and eastern Europe, their distribution was found to extend to the most western part of Europe – Ireland. In Iceland, we found almost the same species spectrum as recorded in the past (see Table 1), with *Echinoparyphium* sp. 2 being a new record, and the presence of *E. recurvatum* was newly confirmed by genetic data from Iceland. Since Iceland lies on the East Atlantic flyway and is a nesting place for birds which overwinter on the British Isles, Norway or other parts [22], the trematode species are shared with continental Europe (*E. recurvatum*, *Ec. revolutum s. str.*, *Echinostoma* sp. IG) and none are endemic to Iceland. *Neopetasiger islandicus* and *Echinoparyphium* sp. 2 using snails of American origin as first intermediate hosts are shared with the North American continent which could be explained by American birds using Iceland as a stopping or staging place when flying to/from their northern nesting places. The records of two known species, *Ec. revolutum* and *E. rubrum* found by us in Alaska (USA) together with the previous records based on DNA sequence data [24, 25, 51, 109, 126, 129], demonstrate that the geographical range of these species is wider than previously known, stretching between the northeast of the USA to the extreme northwest of the North American continent. The results of the phylogenetic analyses of *Echinostoma* spp. are consistent with those in previous studies [46, 48] and confirm the distribution of *Ec*. *revolutum* in North America and *Ec*. *revolutum s*. *str*. in Europe. These closely related species differ not only genetically, but also in morphology of their cercarial stages.

Two species in our material collected in Iceland, namely *N*. *islandicus* from *Gyraulus* cf. *parvus* and *Echinoparyphium* sp. 2 from *Physa acuta* were recorded in both Iceland and North America. Both snail host species are originally non-native to Iceland. *Gyraulus parvus* is native to North America and is also known from Greenland; however, it has been reported to invade freshwaters in central and western Europe [6, 28, 50, 135], and from Iceland it was reported by Meier-Brook [94]. While there are no data on its invasion pathway to Iceland, it is possible that it was introduced into the Icelandic freshwater ecosystems and later became a suitable host for North American trematodes brought with infected migratory birds. Although *N*. *islandicus* was described from an individual of horned grebe *Po*. *auritus* belonging to the isolated Icelandic population [77], this trematode species was recorded in a variety of bird hosts in the southern and northern states of the USA: in *Po. auritus* from Mississippi in 2004, and in the western grebe *Aechmophorus occidentalis* (Lawrence) and the red-necked grebe *Podiceps grisegena* (Boddaert) in North Dakota in 2005 and 2008, respectively ([126], unpublished data). According to Boulet et al. [12] and Kostadinova & Skírnisson [77], the Icelandic population of *Po*. *auritus* is genetically distinct and strongly isolated geographically. Therefore, the species of *N*. *islandicus* was probably introduced to Iceland with its North American bird hosts.

The physid snail *Physa acuta* is native to North America and is currently considered an invasive species globally [28]; it is known to tolerate a wide range of environmental conditions, and it is a rapid coloniser as it can efficiently disperse via water, aquatic birds and mammals [86]. Due to its invasive nature, it has rapidly dispersed within southern and, more recently, in northern Europe [26]. In Iceland, it was introduced more than 40 years ago by spilling snail eggs from aquaria to a ditch in Reykjavík (Skírnisson & Schleich, unpublished). Snails of *P. acuta* were a subject of previous parasitological studies in Iceland ([120], Skírnisson and Schleich, unpublished). However, to date they were not reported as hosts for any trematode intramolluscan stages (sporocysts or rediae), only metacercariae (echinostome and strigeid) encysted in the snails were found in 2007 in Iceland (Skírnisson and Schleich, unpublished). During our study, these snails were found in a single locality, a pond at Nordic House (Table 2) and after examination of almost 700 individuals, infection with cercariae of *Echinoparyphium* sp. 2 was found in 18.9%. Apart from cercariae of *Echinoparyphium* sp. 2, the snails were also infected with echinostome and strigeid (of the genus *Cotylurus*) metacercariae. The relationships between trematodes and invertebrate second intermediate hosts are generally less specific [41] and, thus, it is not surprising that *P*. *acuta* serves as a second intermediate host for metacercariae and likely participates in transmission of the infection to the definitive bird hosts in Iceland. Prior to our study, in Europe *P*. *acuta* was reported as a host for cercariae from the “furcocercous” group in Spain [131] and cercariae of *Fasciola hepatica* L., 1758 in France [27]. However, both cases were not molecularly characterised. Our study reports the third record of *P*. *acuta* as a host for trematodes in Europe with supporting molecular and morphological evidence.

Two species of *Neopetasiger* were recorded in the present study. *Neopetasiger islandicus* was found parasitising *Gyraulus* cf. *parvus* in two localities in Iceland (Table 2). The previous records [47, 77] and our data demonstrate that the distribution of this species in Iceland is associated with the distribution of their definitive hosts – the population of horned grebe *Po*. *auritus* – but also with the distribution of the first intermediate host, i.e. *G.* cf. *parvus* [94]. The second species, *Neopetasiger* sp. 5 was recorded from *Pl*. *planorbis* in Lough Corrib in Ireland. Based on molecular and morphological analyses, this isolate did not match any of the previously described cercariae of *Neopetasiger* spp. in Europe or the genetic sequences published prior to our study. According to the most recent revision of the Echinostomatoidea [126], the genus *Neopetasiger* includes 14 valid species. Out of 14 species, five species have been described and reported in Europe: *N*. *grandivesicularis* (Ishii, 1935), *N*. *islandicus*, *N*. *megacanthus* (Kotlán, 1922), *N*. *neocomense* (Fuhrmann, 1927) and *N*. *pungens* (Linstow, 1894). Life cycles of *N*. *grandivesicularis*, *N*. *islandicus* and *N*. *neocomense* were elucidated and described including morphological descriptions of the larval stages [47, 68, 74]. Additionally, two latter species were molecularly characterised [47]. Therefore, cercariae of *Neopetasiger* sp. 5 may represent either *N*. *megacanthus* or *N*. *pungens* or a new, previously undescribed species. Our study brings the total number of molecularly characterised species within the genus *Neopetasiger* in Europe up to six, whereas, to date, adults of only five species are known. Thus, a higher diversity of *Neopetasiger* in Europe is reported and adults from bird definitive hosts are needed to definitively describe this diversity.

Two species of valve snails *Valvata* (Heterobranchia), *V*. *macrostoma* and *V*. *piscinalis* are known as first intermediate hosts for echinostomes in Europe, including four species of *Echinoparyphium* [37, 54, 58]. Previously, *Valvata macrostoma* was reported as a host for two unidentified species of *Echinoparyphium* in Finland [37], and *V*. *piscinalis* as a host for *E*. *recurvatum* in the UK [58] and *E. mordwilkoi* in Lithuania [54]. Thus far, only one record for *E*. *mordwilkoi* from *V*. *piscinalis* in Lithuania was confirmed by DNA sequences [123]. Here, based on combined genetic and morphological characterisation, we report *Echinoparyphium* sp. 1 from *V*. *macrostoma* in Finland, thus confirming the presence of another species of *Echinoparyphium* parasitising *Valvata* in Europe based on an integrative taxonomic approach.

One of the most interesting findings of our study is the report of an echinostome species that most likely represents a member of a yet unknown genus of the Echinostomatidae. We could not assign Echinostomatidae gen. sp. to any currently known genus within the family based on morphological and molecular genetic analyses. Morphologically, it could potentially belong to *Bashkirovitrema* or *Balfouria*. However, members of neither genus have been reported in Europe thus far. Cercariae of Echinostomatidae gen. sp. show a combination of morphological characters similar to cercariae of *Isthmiophora* spp., *Echinoparyphium* spp. or *Echinostoma* spp. and they could be misidentified when based solely on analyses of cercarial morphology. This once again highlights the importance of DNA sequencing along with morphological characterisation for accurate species identification and evaluation of biodiversity.

Echinostomes have a wide range of first intermediate hosts which includes numerous species of freshwater pulmonate gastropods, with a few species (single species of *Echinostoma*, *Echinoparyphium* and *Neoacanthoparyphium*) parasitising snails belonging to former orthogastropods [126]. The present findings corroborate the intermediate host-use patterns found by Tkach et al. [126]. In the present study, echinostome cercariae were found in 11 species of snails from three families of pulmonate gastropods, Lymnaeidae, Physidae and Planorbidae, and one family of “lower Heterobranchia”, Valvatidae. The number of echinostome species in different hosts did not vary greatly and ranged from one to three species. Only three echinostome species were shared between two snail species, *E*. *recurvatum* and *Echinostoma* sp. IG between *R*. *balthica* and *M*. *glutinosa*, and *Ec*. *revolutum* between *R*. *auricularia* and *S*. *elodes*. In a recent study on echinostomes from sub-Saharan Africa, Laidemitt et al. [84] recovered a high diversity of species which used a wide spectrum of snail hosts, some of their clades using even snails of different genera or families as first intermediate hosts. Compared to the situation near the equator, in the northern latitudes of the northern hemisphere, the spectrum of snail species used as hosts is less diverse, which is mainly connected to the generally lower diversity of free-living biota available as hosts in such regions [139]. Regardless of these differences in snail diversity, for echinostomes globally, it appears that they are a diverse group occurring in a wide range of snail species.

The majority of echinostomes exhibit stenoxenic specificity to their first intermediate hosts [126]. However, the molecular genetic analyses in the present study and in the previous study of Tkach et al. [129] demonstrated that *E. rubrum* is a euryxenous species and utilises snails from at least two families, namely Lymnaeidae (*S. elodes*, present study) and Planorbidae (*Helisoma trivolvis* [129]) as first intermediate hosts. Additionally, this species was reported from *Physa* spp. (Physidae [67]), albeit without DNA sequence confirmation. A more extensive assessment of the snail host range of echinostomes via integrative taxonomy will possibly lead to more discoveries of trematode specificity and transmission pathways, further helping to reveal ecological patterns in these host-parasite interactions.

Out of 12 species of echinostomes found in Europe during this study, four species were reported for the first time, showing that the diversity of these trematodes still remains unsatisfactorily sampled. The position of the species within the phylogenetic tree corresponded to that presented by Tkach et al. [126]; *Hypoderaeum* fell within the clade of *Echinoparyphium*, and the position of *E. aconiatum* still indicates its possible position as a separate genus [126], a state which needs further investigation. Laidemitt et al. [84] in their study on echinostome trematodes from African snails, recorded the same genera (*Echinoparyphium*, *Echinostoma*, *Isthmiophora*, *Patagifer*, *Petasiger*, and *Ribeiroia*) as in the northern hemisphere; however, they contained different species spectra. At the same time, there are few previous studies reporting echinostome species distributions to span the globe, all of them providing DNA sequences: Alberson et al. [2] reported both North and South American haplotypes of *Drepanocephalus auritus* Kudlai, Kostadinova, Pulis, and Tkach, 2015 (formerly reported as *D. spathans* Dietz, 1909) in *Biomphalaria havanensis* L. Pfeiffer, 1839 in catfish aquaculture ponds in Mississippi, most likely due to overlapping feeding ranges of the bird definitive hosts. Furthermore, Georgieva et al. [45] provided the first report of *Ec*. *miyagawai* Ishii 1932 in mallard, *Anas platyrhynchos* L., in New Zealand having previously reported it from the same host, as well as tufted duck, *Aythya fuligula* L. and *P*. *planorbis* snails in Europe [46]. We assume that it is likely that human-caused introductions of waterfowl rather than bird migration led to the introduction of *Ec*. *miyagawai* to New Zealand, where it could possibly adapt to a local intermediate snail host. Interestingly, Laidemitt et al. [84] reasoned that in Africa and South America, there might be similar trematode species using related snail and bird hosts, suggesting the historical connection of the continents as the cause. In the northern hemisphere, there could be a similar situation, or at least it could be assumed that some of the sister species (e.g. *Ec. revolutum*) diversified. The overlap of “European” and “North American” echinostome species was observed only in Iceland; however, there are no data from North Russia to evaluate the species exchange via the Bering Strait or a possible circumpolar distribution of some species, given that the common snail *R. balthica* was recorded in Siberia [133]. Moreover, more data from more southern regions of the northern hemisphere are required to fully decipher species ranges.

It has also been noted that migratory birds are likely to change their migration patterns in response to climate change [60, 63, 140], leading to migration over larger distances with an increased number of stopovers [60] and longer lengths of stay at stopovers [92]. Shifts in species ranges have also been predicted for some snail species, indicating range contractions rather than expansions on both hemispheres [19, 124]. Such changes of parasite host ranges will require more focused investigations in order to better understand the full extent of parasite distributions.

Therefore, further large-scale sampling of echinostomes from the natural snail host populations in areas where birds, their primary hosts, are abundant are needed. And studies providing molecular evaluation of echinostome adults are required to clarify species identity which will shed more light on the species diversity and host associations which can be used in evaluation of parasite latitudinal range shifts. Moreover, based on the distribution of at least two species (e.g. *Echinoparyphium* sp. 2 and *N. islandicus*) extending across both North America and Iceland in the present study, it is clear that large-scale studies are essential for assessing geographical distribution of these parasites.

Although in recent years the family Echinostomatidae has received considerable attention, particularly in molecular genetic studies, there is still a need for such studies to gain data for accurate species identification, especially within the species complexes (“*revolutum*” complex and “*trivolvis*” complex). This should be achieved by following similar guidelines for “best molecular practice” recommended in trematode systematics [9]. DNA barcode sequencing became an essential resource for trematode identification particularly when working with their larval stages. These can be identified reliably to species or genus level via comparison to accurately identified sequence data available in public DNA databases. When publishing new DNA sequence data, it is important to consider previous studies and follow uniform data presentation and subsequent numbering of unidentified species. This will help to avoid misidentification and misinterpretations in later studies and will increase our understanding of the diversity of trematodes.

## Supplementary Materials

The supplementary of this article is available at https://www.parasite-journal.org/10.1051/parasite/2021054 Supplementary Tables *Supplementary Table S1*: Pairwise comparisons of genetic distances of the highlighted clades (see Fig. 1) between *Echinoparyphium* spp. based on *nad*1 sequences.
*Supplementary Table S2*: Pairwise comparisons of genetic distances of the highlighted clades (see Fig. 2) between *Echinostoma* spp. based on *nad*1 sequences.
*Supplementary Table S3*: Pairwise comparisons of genetic distances of the highlighted clades (see Fig. 3) between *Neopetasiger* spp. based on *nad*1 sequences.
*Supplementary Table S4*: Pairwise comparisons of genetic distances of the highlighted clades (see Fig. 4) between the members of the Echinostomatidae based on *28S* sequences.

## Conflict of interest

The authors declare that they have no conflict of interest.
