# Supplementary material for: Diversity of echinostomes (Digenea: Echinostomatidae) in their snail hosts at high latitudes
Source: Parasite. 2021 Jul 28;28:59. doi: 10.1051/parasite/2021054 (PMC8336728; doi:10.1051/parasite/2021054)
Supplement: Supplementary Tables — Supplementary Table S1: Pairwise comparisons of genetic distances of the highlighted clades (see Fig. 1) between Echinoparyphium spp. based on nad1 sequences. Supplementary Table S2: Pairwise comparisons of genetic distances of the highlighted clades (see Fig. 2) between Echinostoma spp. based on nad1 sequences. Supplementary Table S3: Pairwise comparisons of genetic distances of the highlighted clades (see Fig. 3) between Neopetasiger spp. based on nad1 sequences. Supplementary Table S4: Pairwise comparisons of genetic distances of the highlighted clades (see Fig. 4) between the members of the Echinostomatidae based on 28S sequences. [file parasite-28-59-s1.zip › parasite210057-1-olm/Supplementary table S1_genetic distance_Echinoparyphium_spp.pdf]

Supplementary Table S1. Pairwise comparisons of genetic distances of the highlighted clades (see Figure 1) between *Echinoparyphium* spp. based on *nad1* sequences

		**1**	**2**	3	**4**	**5**	**6**	**7**	**8**	**9**	10	**11**	**12**	**13**	**14**	15	16	**17**	18	19	20	21	**22**	**23**	24	**25**	26	27	**28**	**29**	**30**	**31**	**32**	**33**	**34**	35	**36**	**37**
**1**	**AF228 *Echinoparyphium recurvatum***																																					
**2**	**AF256 *Echinoparyphium recurvatum***	0.0																																				
3	AY168940 *Echinoparyphium recurvatum*	1.2	1.2																																			
**4**	**AF229 *Echinoparyphium recurvatum***	0.5	0.5	1.2																																		
**5**	**AF211 *Echinoparyphium recurvatum***	0.2	0.2	1.0	0.2																																	
**6**	**AF222 *Echinoparyphium recurvatum***	0.5	0.5	1.2	0.5	0.2																																
**7**	**AF210 *Echinoparyphium recurvatum***	0.2	0.2	1.0	0.2	0.0	0.2																															
**8**	**AF205 *Echinoparyphium recurvatum***	1.7	1.7	2.4	1.7	1.4	1.7	1.4																														
**9**	**AF220 *Echinoparyphium recurvatum***	1.2	1.2	1.9	1.2	1.0	1.2	1.0	1.0																													
10	KY513266 *Echinoparyphium recurvatum*	1.7	1.7	2.4	1.7	1.4	1.7	1.4	2.4	1.4																												
**11**	**AF254 *Echinoparyphium recurvatum***	1.0	1.0	1.7	1.0	0.7	1.0	0.7	1.7	1.2	0.7																											
**12**	**AF255 *Echinoparyphium recurvatum***	1.0	1.0	1.7	1.0	0.7	1.0	0.7	1.7	1.2	0.7	0.0																										
**13**	**AF204 *Echinoparyphium recurvatum***	2.6	2.6	3.1	2.6	2.4	2.6	2.4	3.4	2.9	2.4	1.7	1.7																									
**14**	**AF241 *Echinoparyphium rubrum***	17.0	17.0	17.5	17.0	17.0	16.8	17.0	18.0	17.5	17.0	16.3	16.3	15.8																								
15	MH369069 *Echinoparyphium* sp. A	17.0	17.0	17.5	17.0	17.0	16.8	17.0	18.0	17.5	17.0	16.3	16.3	15.8	0.0																							
16	MH369107 *Echinoparyphium* sp. Lineage 2	16.8	16.8	17.3	16.8	16.8	16.5	16.8	17.7	17.3	16.8	16.1	16.1	15.6	0.2	0.2																						
**17**	**AF244 *Echinoparyphium rubrum***	16.5	16.5	17.0	16.5	16.5	16.3	16.5	17.5	17.0	16.5	15.8	15.8	15.3	0.7	0.7	0.5																					
18	MH369130 *Echinoparyphium* sp. Lineage 3/4	16.3	16.3	16.8	16.3	16.3	16.1	16.3	17.3	16.8	16.3	15.6	15.6	15.1	1.7	1.7	1.4	1.9																				
19	MH369046 *Echinoparyphium* sp. 1A	16.3	16.3	16.8	16.3	16.3	16.1	16.3	17.3	16.8	16.1	15.6	15.6	15.1	1.2	1.2	1.0	1.0	1.0																			
20	MH368958 *Hypoderaeum* sp. Lineage 1	16.1	16.1	16.5	16.1	16.1	15.8	16.1	17.0	16.5	16.0	15.3	15.3	14.9	1.4	1.4	1.2	1.2	1.2	0.2																		
21	MH369109 *Echinoparyphium* sp. E	16.0	16.0	16.5	16.0	16.0	15.8	16.0	16.8	16.5	23.3	15.3	15.3	15.1	1.5	1.5	1.2	1.2	1.2	0.2	0.0																	
**22**	**AF420 *Echinoparyphium* sp. 2**	23.3	23.3	23.5	23.3	23.3	23.0	23.3	23.7	23.0	23.3	23.0	23.0	22.3	16.8	16.8	17.0	16.8	16.3	16.1	16.3	16.3																
**23**	**AF423 *Echinoparyphium* sp. 2**	23.3	23.3	23.5	23.3	23.3	23.0	23.3	23.7	23.0	23.0	23.0	23.0	22.3	16.8	16.8	17.0	16.8	16.3	16.1	16.3	16.3	0.0															
24	MH369080 *Hypoderaeum Lineage* 2	23.0	23.0	23.3	23.0	23.0	22.8	23.0	23.5	22.8	23.0	22.8	22.8	22.1	16.5	16.5	16.8	16.5	16.1	15.8	16.1	16.0	0.2	0.2														
**25**	**AF421 *Echinoparyphium* sp. 2**	22.4	22.4	22.6	22.4	22.4	22.1	22.4	23.3	22.6	22.4	22.1	22.1	21.9	16.8	16.8	17.1	16.8	16.3	16.1	16.3	16.3	2.2	2.2	1.9													
26	MH369047 *Echinoparyphium* sp. 1A	22.3	22.3	22.5	22.3	22.3	22.1	22.3	22.8	22.1	22.3	22.1	22.1	21.3	16.3	16.3	16.5	16.3	15.8	15.6	15.8	15.8	1.0	1.0	0.7	2.2												
27	MH369158 *Echinoparyphium* sp. Lineage 3/4	22.5	22.5	22.8	22.5	22.5	22.3	22.5	23.0	22.3	22.5	22.3	22.3	21.6	16.5	16.5	16.8	16.5	16.1	15.8	16.1	16.0	0.7	0.7	0.5	2.4	0.2											
**28**	**AF251 *Echinoparyphium* sp. 1**	23.5	23.5	23.7	23.5	23.5	23.3	24.2	24.2	24.0	23.7	23.3	23.3	22.5	18.2	18.2	18.5	18.0	18.9	18.5	18.5	18.5	20.9	20.9	20.6	20.4	20.9	21.1										
**29**	**AF252 *Echinoparyphium* sp. 1**	23.5	23.5	23.7	23.5	23.5	23.3	23.5	24.2	24.0	23.7	23.3	23.3	22.5	18.2	18.2	18.5	18.0	18.9	18.5	18.5	18.5	20.9	20.9	20.6	20.4	20.9	21.1	0.0									
**30**	**AF253 *Echinoparyphium* sp. 1**	23.5	23.5	23.7	23.5	23.5	23.3	23.5	24.2	24.0	23.7	23.3	23.3	22.5	18.2	18.2	18.5	18.0	18.9	18.5	18.5	18.5	20.9	20.9	20.6	20.4	20.9	21.1	0.0	0.0								
**31**	**AF225 *Echinoparyphium aconiatum***	23.3	23.3	24.0	23.5	23.3	23.0	23.3	24.2	23.7	23.5	23.0	23.0	22.5	19.4	19.4	19.2	19.2	18.7	18.7	18.5	18.8	21.6	21.6	21.3	20.4	21.1	20.9	19.4	19.4	19.4							
**32**	**AF226 *Echinoparyphium aconiatum***	23.3	23.3	24.0	23.5	23.3	23.0	23.3	24.2	23.7	23.5	23.0	23.0	22.5	19.4	19.4	19.2	19.2	18.7	18.7	18.5	18.8	21.6	21.6	21.3	20.4	21.1	20.9	19.4	19.4	19.4	0.0						
**33**	**AF275 *Echinoparyphium aconiatum***	23.3	23.3	24.0	23.5	23.3	23.0	23.3	24.2	23.7	23.5	23.0	23.0	22.5	19.4	19.4	19.2	19.2	18.7	18.7	18.5	18.8	21.6	21.6	21.3	20.4	21.1	20.9	19.4	19.4	19.4	0.0	0.0					
**34**	**AF273 *Echinoparyphium aconiatum***	23.3	23.3	24.0	23.5	23.3	23.0	23.3	24.2	23.7	23.5	23.0	23.0	22.5	19.4	19.4	19.2	19.2	18.7	18.7	18.5	18.8	21.6	21.6	21.3	20.4	21.1	20.9	19.4	19.4	19.4	0.0	0.0	0.0				
35	AY168947 *Echinoparyphium aconiatum*	23.0	23.0	23.7	23.3	23.0	22.8	23.0	24.0	23.5	23.3	22.8	22.8	22.3	19.2	19.2	18.9	18.9	18.5	18.5	18.2	18.5	21.3	21.3	21.1	20.2	20.9	20.6	19.2	19.2	19.2	0.2	0.2	0.2	0.2			
**36**	**AF227 *Echinoparyphium aconiatum***	23.0	23.0	23.7	23.3	23.0	22.8	23.0	23.5	23.5	23.3	22.8	22.8	22.3	19.2	19.2	18.9	18.9	18.5	18.5	18.2	18.3	21.6	21.6	21.1	20.4	21.1	20.9	19.4	19.4	19.4	1.4	1.4	1.4	1.4	1.2		
**37**	**AF274 *Echinoparyphium aconiatum***	23.3	23.3	24.0	23.5	23.3	23.0	23.3	23.7	23.7	23.5	23.0	23.0	22.5	19.9	19.9	19.7	19.2	19.2	18.7	18.5	18.5	20.9	20.9	20.6	19.7	20.9	20.6	19.2	19.2	19.2	1.4	1.4	1.4	1.4	1.2	1.0	
