# Supplementary material for: Diversity of echinostomes (Digenea: Echinostomatidae) in their snail hosts at high latitudes
Source: Parasite. 2021 Jul 28;28:59. doi: 10.1051/parasite/2021054 (PMC8336728; doi:10.1051/parasite/2021054)
Supplement: Supplementary Tables — Supplementary Table S1: Pairwise comparisons of genetic distances of the highlighted clades (see Fig. 1) between Echinoparyphium spp. based on nad1 sequences. Supplementary Table S2: Pairwise comparisons of genetic distances of the highlighted clades (see Fig. 2) between Echinostoma spp. based on nad1 sequences. Supplementary Table S3: Pairwise comparisons of genetic distances of the highlighted clades (see Fig. 3) between Neopetasiger spp. based on nad1 sequences. Supplementary Table S4: Pairwise comparisons of genetic distances of the highlighted clades (see Fig. 4) between the members of the Echinostomatidae based on 28S sequences. [file parasite-28-59-s1.zip › parasite210057-1-olm/Supplementary table S2_genetic distance_EchinostomaCorr.pdf]

Supplementary Table S2. Pairwise comparisons of genetic distances of the highlighted clades (see Figure 2) between *Echinostoma* spp. based on *nad1* sequences

		1	**2**	**3**	4	**5**	**6**	7	**8**	9	10	11	**12**	13	14	**15**	**16**	17	**18**	19	**20**	21	**22**
**1**	**AF214 *Echinostoma revolutum s.str.***																						
**2**	**AF219 *Echinostoma revolutum s.str.***	0.0																					
**3**	**AF215 *Echinostoma revolutum s.str.***	0.7	0.7																				
4	LC224099 *Echinostoma revolutum* ***s.str.***	0.7	0.7	0.9																			
**5**	**AF216 *Echinostoma revolutum s.str.***	1.2	1.2	1.4	1.4																		
**6**	**AF217 *Echinostoma revolutum s.str.***	1.2	1.2	1.4	1.4	0.0																	
7	KC618451 *Echinostoma revolutum* ***s.str.***	0.9	0.9	1.2	1.2	1.2	1.2																
**8**	**AF206 *Echinostoma revolutum s.str.***	1.2	1.2	1.4	1.4	1.4	1.4	1.6															
9	KP065655 *Echinostoma revolutum* ***s.str.***	0.9	0.9	1.2	1.2	1.2	1.2	1.4	0.7														
10	**AF237 *Echinostoma revolutum***	5.8	5.8	5.6	5.3	5.6	5.6	5.8	6.0	5.3													
11	GQ463090 *Echinostoma revolutum*	6.0	6.0	5.8	5.6	5.8	5.8	6.0	6.3	5.6	0.2												
**12**	**AF236 *Echinostoma revolutum***	5.8	5.8	5.6	5.3	5.6	5.6	5.8	6.0	5.3	0.0	0.2											
13	JQ670862 *Echinostoma revolutum*	6.0	6.0	5.8	5.6	5.8	5.8	6.0	6.3	5.6	0.7	0.9	0.7										
14	MH369198 *Echinostoma trivolvis* Lineage A	6.0	6.0	5.8	5.6	5.8	5.8	6.0	6.3	5.6	0.7	0.9	0.7	0.5									
**15**	**AF235 *Echinostoma revolutum***	5.3	5.3	5.1	4.9	5.1	5.1	5.3	5.6	4.9	0.7	0.9	0.7	0.9	0.9								
**16**	**AF232 *Echinostoma nasincovae***	12.8	12.8	12.6	13.0	12.3	12.3	12.8	13.0	12.3	12.3	12.6	12.3	13.0	13.0	12.6							
17	KP065676 *Echinostoma nasincovae*	12.8	12.8	12.6	12.8	12.3	12.3	12.8	13.0	12.3	11.9	12.1	11.9	12.6	12.6	12.1	0.7						
**18**	**AF218 *Echinostoma* sp. IG**	18.1	18.1	18.4	18.4	18.1	18.1	18.4	18.4	17.7	18.6	18.8	18.6	18.6	18.4	17.9	19.5	19.8					
19	KC618448 *Echinostoma* sp. IG	18.1	18.1	18.4	18.4	18.1	18.1	18.4	18.4	17.7	19.1	19.3	19.1	19.1	18.8	18.4	19.5	19.8	0.5				
**20**	**AF221 *Echinostoma* sp. IG**	18.1	18.1	18.4	18.4	18.1	18.1	18.4	18.4	17.7	19.1	19.3	19.1	19.1	18.8	18.4	19.5	19.8	0.7	0.2			
21	KC618449 *Echinostoma* sp. IG	17.9	17.9	18.1	18.1	17.9	17.9	18.1	18.1	17.4	18.8	19.1	18.8	18.8	18.6	18.1	19.3	19.5	0.7	0.2	0.5		
**22**	**AF231 *Echinostoma* sp. IG**	18.4	18.4	18.6	18.4	18.4	18.4	18.1	18.6	18.4	19.3	19.5	19.3	19.3	19.1	18.6	20.2	20.0	1.2	0.7	0.9	0.9	
