# Supplementary material for: Diversity of echinostomes (Digenea: Echinostomatidae) in their snail hosts at high latitudes
Source: Parasite. 2021 Jul 28;28:59. doi: 10.1051/parasite/2021054 (PMC8336728; doi:10.1051/parasite/2021054)
Supplement: Supplementary Tables — Supplementary Table S1: Pairwise comparisons of genetic distances of the highlighted clades (see Fig. 1) between Echinoparyphium spp. based on nad1 sequences. Supplementary Table S2: Pairwise comparisons of genetic distances of the highlighted clades (see Fig. 2) between Echinostoma spp. based on nad1 sequences. Supplementary Table S3: Pairwise comparisons of genetic distances of the highlighted clades (see Fig. 3) between Neopetasiger spp. based on nad1 sequences. Supplementary Table S4: Pairwise comparisons of genetic distances of the highlighted clades (see Fig. 4) between the members of the Echinostomatidae based on 28S sequences. [file parasite-28-59-s1.zip › parasite210057-1-olm/Supplementary table S3_genetic distance_Neopetasiger_spp.pdf]

Supplementary Table S3. Pairwise comparisons of genetic distances of the highlighted clades (see Figure 3) between *Neopetasiger* spp. based on *nad1* sequences.

		**1**	**2**	3	**4**	5	**6**
**1**	**AF416_*Neopetasiger islandicus***						
**2**	**AF415 *Neopetasiger islandicus***	0.0					
3	JQ425588 *Petasiger islandicus*	0.2	0.2				
**4**	**AF418 *Neopetasiger islandicus***	0.0	0.0	0.2			
5	KT831342 *Neopetasiger islandicus*	3.2	3.2	3.5	3.2		
**6**	**AF233 *Neopetasiger* sp. 5**	19.4	19.4	19.7	19.4	19.2	
