# Supplementary material for: Diversity of echinostomes (Digenea: Echinostomatidae) in their snail hosts at high latitudes
Source: Parasite. 2021 Jul 28;28:59. doi: 10.1051/parasite/2021054 (PMC8336728; doi:10.1051/parasite/2021054)
Supplement: Supplementary Tables — Supplementary Table S1: Pairwise comparisons of genetic distances of the highlighted clades (see Fig. 1) between Echinoparyphium spp. based on nad1 sequences. Supplementary Table S2: Pairwise comparisons of genetic distances of the highlighted clades (see Fig. 2) between Echinostoma spp. based on nad1 sequences. Supplementary Table S3: Pairwise comparisons of genetic distances of the highlighted clades (see Fig. 3) between Neopetasiger spp. based on nad1 sequences. Supplementary Table S4: Pairwise comparisons of genetic distances of the highlighted clades (see Fig. 4) between the members of the Echinostomatidae based on 28S sequences. [file parasite-28-59-s1.zip › parasite210057-1-olm/Supplementary table S4_genetic distance_EchinostomatidaeCorr.pdf]

Supplementary Table S4. Pairwise comparisons of genetic distances of the highlighted clades (see Figure 4) between the members of the Echinostomatidae based on *28S* sequences

		1	**2**	**3**	4	**5**	**6**	7	8	9	**10**	11	**12**	13	**14**	15	**16**	**17**	18	**19**	**20**	**21**	22	**23**	24	**25**	**26**	27	**28**	29	30	31	32	33	**34**	35	36	37	38	39	**40**	41	**42**	43	44	45	46
1	JX262943 *Echinoparyphium rubrum*																																														
**2**	**AF241 *Echinoparyphium rubrum***	0.0																																													
**3**	**AF244 *Echinoparyphium rubrum***	0.1	0.1																																												
4	KY436409 *Echinoparyphium poulini*	0.2	0.2	0.3																																											
**5**	**AF204 *Echoparyphium recurvatum***	0.1	0.1	0.2	0.3																																										
**6**	**AF254 *Echoparyphium recurvatum***	0.1	0.1	0.2	0.3	0.0																																									
7	KT956913 *Echinoparyphium recurvatum*	0.1	0.1	0.2	0.3	0.0	0.0																																								
8	AF184260 *Echinoparyphium cinctum*	0.1	0.1	0.2	0.3	0.0	0.0	0.0																																							
9	KY436410 *Echinoparyphium ellisi*	0.2	0.2	0.3	0.4	0.1	0.1	0.1	0.1																																						
**10**	**AF420 *Echinoparyphium* sp. 2**	0.2	0.2	0.3	0.4	0.1	0.1	0.1	0.1	0.0																																					
11	KT956918 *Hypoderaeum conoideum*	0.8	0.8	0.9	1.0	0.7	0.7	0.7	0.7	0.6	0.6																																				
**12**	**AF261 *Hypoderaeum conoideum***	0.8	0.8	0.9	1.0	0.7	0.7	0.7	0.7	0.6	0.6	0.0																																			
13	KJ542642 *Echinoparyphium mordvilkowi*	1.7	1.7	1.8	1.8	1.6	1.6	1.6	1.6	1.5	1.5	1.9	1.9																																		
**14**	**AF252 *Echinparyphium* sp. 1**	1.4	1.4	1.5	1.6	1.3	1.3	1.3	1.3	1.2	1.2	1.7	1.7	0.6																																	
15	KT956912 *Echinoparyphium aconiatum*	1.1	1.1	1.2	1.3	1.1	1.1	1.1	1.1	1.0	1.0	1.4	1.4	1.8	1.7																																
**16**	**AF227 *Echinoparyphium aconiatum***	1.1	1.1	1.2	1.3	1.1	1.1	1.1	1.1	1.0	1.0	1.4	1.4	1.8	1.7	0.0																															
**17**	**AF273 *Echinoparyphium aconiatum***	1.1	1.1	1.2	1.3	1.1	1.1	1.1	1.1	1.0	1.0	1.4	1.4	1.8	1.7	0.0	0.0																														
18	KT956921 *Moliniella anceps*	2.6	2.6	2.6	2.8	2.6	2.6	2.6	2.6	2.5	2.5	2.7	2.7	3.3	3.1	2.7	2.7	2.7																													
**19**	**AF230 *Moliniella anceps***	2.6	2.6	2.6	2.8	2.6	2.6	2.6	2.6	2.5	2.5	2.7	2.7	3.3	3.1	2.7	2.7	2.7	0.0																												
**20**	**AF260 Echinostomatidae gen. sp.**	2.4	2.4	2.5	2.4	2.2	2.3	2.3	2.3	2.2	2.2	2.5	2.5	3.1	2.8	2.3	2.3	2.3	1.4	1.4																											
**21**	**AF258 Echinostomatidae gen. sp.**	2.4	2.4	2.5	2.4	2.2	2.3	2.3	2.3	2.2	2.2	2.5	2.5	3.1	2.8	2.3	2.3	2.3	1.4	1.4	0.0																										
22	KT956915 *Echinostoma revolutum*	3.8	3.8	3.8	3.8	3.7	3.7	3.7	3.7	3.6	3.6	3.5	3.5	4.0	3.7	3.4	3.4	3.4	3.5	3.5	3.8	3.8																									
**23**	**AF235 *Echinostoma revolutum***	3.8	3.8	3.8	3.8	3.7	3.7	3.7	3.7	3.6	3.6	3.5	3.5	4.0	3.7	3.4	3.4	3.4	3.5	3.5	3.8	3.8	0.0																								
24	KP065596 *Echinostoma revolutum s.str.*	3.7	3.7	3.7	3.7	3.6	3.6	3.6	3.6	3.5	3.5	3.4	3.4	3.9	3.6	3.3	3.3	3.3	3.6	3.6	3.7	3.7	0.4	0.4																							
**25**	**AF206 *Echinostoma revolutum s.str.***	3.7	3.7	3.7	3.7	3.6	3.6	3.6	3.6	3.5	3.5	3.4	3.4	3.9	3.6	3.3	3.3	3.3	3.6	3.6	3.7	3.7	0.4	0.4	0.0																						
**26**	**AF218 *Echinostoma* sp. IG**	3.5	3.5	3.5	3.5	3.4	3.4	3.4	3.4	3.3	3.3	3.3	3.3	4.0	3.6	3.2	3.2	3.2	3.6	3.6	3.7	3.7	1.4	1.4	1.3	1.3																					
27	KP065606 *Echinostoma* sp. IG	3.5	3.5	3.5	3.5	3.4	3.4	3.4	3.4	3.3	3.3	3.3	3.3	4.0	3.6	3.2	3.2	3.2	3.6	3.6	3.7	3.7	1.4	1.4	1.3	1.3	0.0																				
**28**	**AF231 *Echinostoma* sp. IG**	3.5	3.5	3.5	3.5	3.4	3.4	3.4	3.4	3.3	3.3	3.3	3.3	4.0	3.6	3.2	3.2	3.2	3.6	3.6	3.7	3.7	1.4	1.4	1.3	1.3	0.0	0.0																			
29	KT956916 *Echinostoma miyagawai*	3.2	3.2	3.2	3.2	3.1	3.1	3.1	3.1	3.0	3.0	2.9	2.9	3.3	3.1	2.8	2.8	2.8	3.1	3.1	3.2	3.2	0.9	0.9	0.8	0.8	1.1	1.1	1.1																		
30	KP065604 *Echinostoma paraulum*	3.1	3.1	3.1	3.1	3.0	3.0	3.0	3.0	2.9	2.9	2.8	2.8	3.1	2.8	2.7	2.7	2.7	3.0	3.0	3.1	3.1	0.9	0.9	0.8	0.8	1.1	1.1	1.1	0.3																	
31	AY222246 *Echinostoma trivolvis*	3.4	3.2	3.4	3.4	3.3	3.3	3.3	3.3	3.3	3.3	3.2	3.2	3.6	3.3	3.1	3.1	3.1	3.3	3.3	3.4	3.4	1.2	1.2	1.1	1.1	1.4	1.4	1.4	0.6	0.5																
32	EU025867 *Echinostoma paraensei*	3.3	3.3	3.3	3.3	3.3	3.3	3.3	3.3	3.2	3.2	2.9	2.9	4.5	3.3	3.0	3.0	3.0	3.1	3.1	3.3	3.3	1.0	1.0	1.1	1.1	1.1	1.1	1.1	0.5	0.4	0.4															
33	KP065603 *Echinostoma nasincovae*	3.2	3.2	3.2	3.2	3.1	3.1	3.1	3.1	3.0	3.0	2.9	2.9	3.3	3.1	2.8	2.8	2.8	3.1	3.1	3.2	3.2	1.0	1.0	0.9	0.9	1.1	1.1	1.1	0.4	0.4	0.4	0.4														
**34**	**AF232 *Echinostoma nasincovae***	3.2	3.2	3.2	3.2	3.1	3.1	3.1	3.1	3.0	3.0	2.9	2.9	3.3	3.1	2.8	2.8	2.8	3.1	3.1	3.2	3.2	1.0	1.0	0.9	0.9	1.1	1.1	1.1	0.4	0.4	0.4	0.4	0.0													
35	KY436407 *Echinostoma novaezealandense*	3.3	3.3	3.3	3.3	3.2	3.2	3.2	3.2	3.1	3.1	3.0	3.0	3.3	3.0	2.9	2.9	2.9	3.0	3.0	3.1	3.1	1.1	1.1	1.1	1.1	1.4	1.4	1.4	0.6	0.5	0.7	0.6	0.6	0.6												
36	MK482501 *Echinostoma caproni*	3.1	3.1	3.1	3.1	3.0	3.0	3.0	3.0	2.9	2.9	3.0	3.0	3.3	3.0	2.7	2.7	2.7	2.8	2.8	2.9	2.9	1.2	1.2	1.1	1.1	1.3	1.3	1.3	0.6	0.5	0.9	0.8	0.6	0.6	0.7											
37	KP065592 *Echinostoma bolschewense*	3.1	3.1	3.1	3.1	3.0	3.0	3.0	3.0	2.9	2.9	3.0	3.0	3.6	3.3	2.8	2.8	2.8	2.8	2.8	2.9	2.9	1.6	1.6	1.5	1.5	1.3	1.3	1.3	0.0	0.9	1.1	1.0	1.0	1.0	0.9	0.7										
38	KT956925 *Neopetasiger* sp.	6.0	6.0	5.9	6.2	6.1	6.1	6.1	6.1	6.2	6.2	6.1	6.1	6.6	6.4	5.8	5.8	5.8	6.0	6.0	6.4	6.4	6.4	6.4	6.4	6.4	6.3	6.3	6.3	6.0	6.0	6.4	6.3	6.1	6.1	6.1	5.9	6.1									
39	KM191807 *Petasiger* sp. 4	6.0	6.0	5.9	6.2	6.1	6.1	6.1	6.1	6.2	6.2	6.1	6.1	6.6	6.4	5.8	5.8	5.8	6.0	6.0	6.4	6.4	6.4	6.4	6.4	6.4	6.3	6.3	6.3	6.0	6.0	6.4	6.3	6.1	6.1	6.1	5.9	6.1	0.0								
**40**	**AF233 *Neopetasiger* sp. 5**	6.1	6.1	6.0	6.1	6.2	6.2	6.2	6.2	6.3	6.3	6.3	6.3	6.8	6.7	5.8	5.8	5.8	6.2	6.2	6.2	6.2	6.5	6.5	6.5	6.5	6.4	6.4	6.4	6.0	6.0	6.3	6.3	6.2	6.2	6.2	5.7	5.9	2.6	2.6							
41	KM191802 *Petasiger* sp. 2	5.3	5.3	5.2	5.5	5.4	5.4	5.4	5.4	5.5	5.5	5.4	5.4	5.8	5.7	4.9	4.9	4.9	5.2	5.2	5.4	5.4	5.9	5.9	5.8	5.8	5.6	5.6	5.6	5.2	5.2	5.6	5.6	5.4	5.4	5.5	5.1	5.3	2.5	2.5	1.5						
**42**	**AF415 *Neopetasiger islandicus***	5.0	5.0	4.9	5.2	5.1	5.1	5.1	5.1	5.2	5.2	5.1	5.1	5.7	5.6	4.9	4.9	4.9	5.1	5.1	5.5	5.5	5.8	5.8	5.8	5.8	5.7	5.7	5.7	5.3	5.4	5.7	5.6	5.5	5.5	5.5	5.2	5.4	2.1	2.1	1.5	0.9					
43	KT956924 *Neopetasiger islandicus*	5.0	5.0	4.9	5.2	5.1	5.1	5.1	5.1	5.2	5.2	5.1	5.1	5.7	5.6	4.9	4.9	4.9	5.1	5.1	5.5	5.5	5.8	5.8	5.8	5.8	5.7	5.7	5.7	5.3	5.4	5.7	5.6	5.5	5.5	5.5	5.2	5.4	2.1	2.1	1.5	0.9	0.0				
44	JQ425592 *Petasiger islandicus*	5.0	5.0	4.9	5.2	5.1	5.1	5.1	5.1	5.2	5.2	5.1	5.1	5.7	5.6	4.9	4.9	4.9	5.1	5.1	5.5	5.5	5.8	5.8	5.8	5.8	5.7	5.7	5.7	5.3	5.4	5.7	5.6	5.5	5.5	5.5	5.2	5.4	2.1	2.1	1.5	0.9	0.0	0.0			
45	KM191804 *Petasiger* sp. 3	5.2	5.2	5.1	5.4	5.3	5.3	5.3	5.3	5.4	5.4	5.3	5.3	5.9	5.8	4.9	4.9	4.9	5.2	5.2	5.4	5.4	5.7	5.7	5.7	5.7	5.6	5.6	5.6	5.2	5.3	5.6	5.6	5.4	5.4	5.4	5.1	5.3	2.4	2.4	1.6	1.3	0.6	0.6	0.6		
46	KM191800 *Petasiger* sp. 1	5.0	5.0	4.9	5.2	5.1	5.1	5.1	5.1	5.2	5.2	5.1	5.1	5.7	5.6	4.7	4.7	4.7	5.1	5.1	5.3	5.3	5.8	5.8	5.7	5.7	5.6	5.6	5.6	5.1	5.2	5.4	5.5	5.3	5.3	5.4	5.0	5.2	2.3	2.3	1.4	1.1	0.6	0.6	0.6	0.7	
